# Proactive fault prediction in marine diesel engines using multivariate machine learning

**DOI:** 10.1038/s41598-026-40979-5

**Published:** 2026-03-20

**Authors:** Miral Michel, Ahmed Mehanna, Sherine Nagy Saleh, Ahmed S. Shehata

**Affiliations:** 1 Marine and Offshore Engineering Department, College of Engineering and Technology, Arab Academy for Science, Technology and Maritime Transport, Alexandria, Egypt; 2 Computer Engineering Department, College of Engineering and Technology, Arab Academy for Science, Technology and Maritime Transport, Alexandria, Egypt

**Keywords:** Shipping, Marine engines, Proactive maintenance, Failure prediction and classification, Smart ship, Energy science and technology, Engineering, Mathematics and computing

## Abstract

Ocean shipping is the backbone of international trade contributing to global economic growth. Consequently, ensuring that ships operate in an energy-efficient manner is crucial to a more sustainable global transportation. Engine failures in these contexts can lead to severe consequences including compromised safety, operational disruptions, and substantial economic losses ranging between 10% and 30% of total operating costs due to unscheduled maintenance. The proposed research integrates marine diesel engines diagnostics with machine learning (ML) algorithms to develop an advanced proactive maintenance strategy to anticipate engine performance trends and proactively identify potential faults before they escalate. Employing an experimental approach on a 4-stroke diesel engine, the controlled simulations were conducted to replicate various failure scenarios to collect data and capture crucial metrics such as temperatures across cylinders, vibrations along axes, and fluctuations in cooling water temperatures. The data were analysed using advanced ML algorithms aimed at enhancing the accuracy and reliability of future fault prediction, by employing a multivariate convolutional long short-term memory (ConvLSTM) model tailored for time series analysis, and a classification model using a random forest (RF) classifier. As a result, the ConvLSTM model decreased the RMSE by 15.4453% compared to decision tree regression models, while the RF classifier achieved an accuracy of 82.168%.

## Introduction

**Fig. 1:**
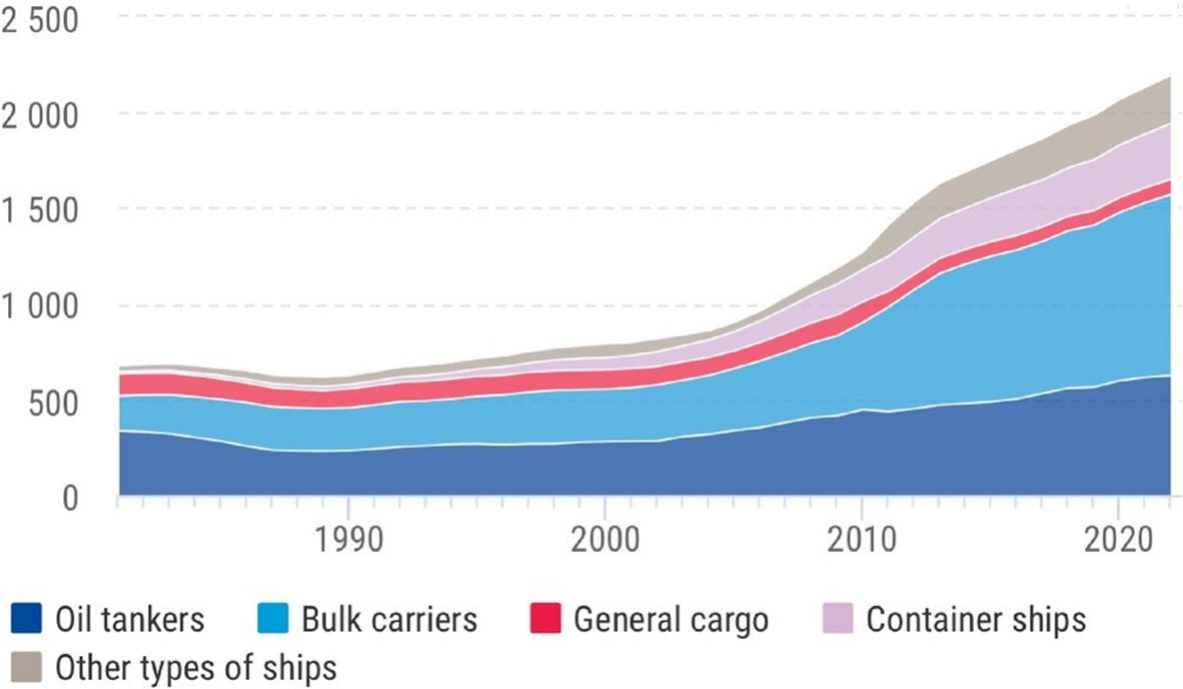
Global merchant fleet distribution by vessel type expressed in millions of deadweight tons (DWT) over time, source: UNCTADstat^2^; Clarksons Research.

According to United Nations (UN) Conference on Trade and Development (UNCTAD) in 2023^1^, the world’s merchant fleet comprises approximately 105,500 vessels actively contributing to international trade. When expressed in terms of carrying capacity, the fleet composition by vessel type is detailed in Fig. 1, highlighting long-term changes in the global shipping industry.

Maritime shipping serves as the backbone of logistics and global supply chain connecting global markets, ensuring efficient transportation of cargo, as well as fostering global economies^2^. Given the size of the maritime industry, the importance of maintenance can’t be emphasized enough as it directly affects the safety of shipping operations and thus their efficiency and profitability. Carrying out regular maintenance tasks can also increase a vessel’s lifespan, lower operating costs, and avoid machinery breakdown which ensure that vessels remain in optimal working conditions.

A proper maintenance schedule of ships’ machinery can also contribute to the reduction of vessels’ environmental footprint by reducing energy consumption and consequently the resulting harmful emissions^3^. Despite being responsible for 90% of world trade, the shipping industry is considered the greatest contributor to emissions by accounting for 1076 million tonnes of carbon dioxide (CO_2_) in 2018. Major efforts are in place by the International Maritime Organization (IMO) to limit greenhouse gas emissions (GHGs) from ships by at least 40% by 2030. The adoption of these strategies comes in compliance with the UN’s sustainable development goal 13 “Take urgent action to combat climate change and its impacts”^4^.

The quantity and type of emissions generated by marine engines are highly dependent on the engine’s operating conditions; various modes of engine failure or inefficient operation such as a maladjustment in combustion parameters can contribute greatly to the formation of nitrogen oxides (NO_x_) and particulate matter (PM)^5^. Proper maintenance practices can ensure that a ship’s machinery remains in desired operating conditions and reduce downtime which can lead to further repair and replacement costs. Furthermore, implementing a proper maintenance strategy is crucial for cost management. A survey by the European Commission indicates that around 33% of maintenance costs are lost due to the improper selection of maintenance activities^6^. Proper maintenance of engines and equipment within a ship’s mechanism is crucial for reducing emissions and fuel usage. By ensuring optimal performance and minimizing fuel consumption, maintenance practices contribute to a greener and more sustainable maritime industry^7^. Without reliable and well-maintained vessels, the efficient movement of goods and commodities across the world would be severely hindered.

Among the key components of a ship, diesel engines stand out as the most critical. Their maintenance is of utmost importance to ensure smooth operations, minimize disruptions, and optimize fuel efficiency. Ensuring the reliability and efficiency of maritime fleets, through rigorous maintenance practices, is essential for maintaining uninterrupted global supply chains, stabilizing market dynamics, and sustaining economic growth on a global scale^8^. The integration of artificial intelligence (AI)-driven solutions into existing maintenance frameworks promises not only to streamline decision-making processes but also to drive significant improvements in operational efficiency and profitability across the maritime sector. Furthermore, the imperative of maintenance extends beyond operational efficiency to encompass environmental sustainability where effective maintenance practices play a pivotal role in reducing emissions and conserving fuel resources^9^. By ensuring optimal engine performance and minimizing environmental impact, maintenance initiatives align with global efforts to combat climate change and promote sustainable development goals (SDGs)^10^.

Ships, valued at more than $150 million each to construct, collectively contribute around $500 billion in annual revenues, making up a significant 5% of the global economic output. The infrastructure of ports and fleets plays a pivotal role in reducing transportation costs, stimulating economic growth, and supporting the intricate global logistics network^11^. The maintenance of commercial ships is paramount to ensuring operational efficiency and safety at sea. Various maintenance strategies are employed, each presenting unique benefits and challenges. Reactive maintenance responds to breakdowns but entails significant costs and operational disruptions. Corrective maintenance addresses emerging faults promptly but may escalate long-term expenses. Preventive maintenance involves routine scheduled checks to anticipate deterioration, while proactive maintenance harnesses data analytics to foresee potential issues, thereby minimizing downtime and costs. Proactive maintenance takes a preemptive stance by systematically inspecting and addressing potential issues, thereby ensuring optimal equipment reliability and safety. Implementing proactive maintenance in maritime operations demands upfront investments in advanced technology and comprehensive training. However, the long-term benefits are substantial, including enhanced equipment reliability, minimized downtime, and improved safety standards^12^.

Maintenance in the marine transport industry is a multifaceted and critically important aspect that involves a wide array of techniques and approaches^13^. Despite its focus, current maintenance practices often fall short of achieving optimal outcomes, leading to increased incidents of failures and downtime that disrupt the efficient operation of ships and pose potential safety hazards^14^. These shortcomings are intensified by the challenges inherent in addressing the harsh marine environment, rigorous operational demands, and the necessity to comply with stringent regulatory requirements^15^. As a result, existing maintenance strategies struggle to consistently meet the industry’s high standards of reliability, efficiency, and environmental sustainability. The integration of AI and machine learning (ML) algorithms into the maritime industry presents a pivotal opportunity but also poses significant challenges. ML processes enable models to detect and learn from faults, thereby enabling proactive maintenance strategies that anticipate future failures based on data such as usage patterns, operational routines, and other relevant parameters. However, accurately assessing the severity of anomalies remains a fundamental challenge, even for human evaluators, due to varying expertise and subjective interpretations^16^.

To confront these pressing issues, comprehensive studies and the development of standardized guidelines tailored to the unique characteristics and challenges of maritime transport are essential^17^. Efforts to enhance engine efficiency and reduce emissions, particularly in diesel engines renowned for their higher thermal efficiency but significant NO_x_ emissions and smoke, are crucial for sustainable maritime operations^18^. In response to the energy crisis and increasing environmental regulations, advancements in engine technology are focusing on improving fuel efficiency and reducing emissions. Furthermore, the deployment of AI and ML technologies in maintenance operations support condition-based monitoring, performance optimization, and fleet management strategies that enhance operational efficiency and life cycle cost management. By leveraging big data analytics and real-time monitoring capabilities, ship operators can make informed decisions that optimize vessel performance, reduce maintenance costs, and extend equipment lifespan. In recent years, extensive research has been conducted to explore the outcomes of maintenance practices in the maritime industry. These studies have employed various methodologies to investigate the effectiveness of different maintenance approaches and their impact on efficiency and performance. Condition-based maintenance (CBM) utilizes real-time monitoring and data analysis to enable the early detection of potential faults and failures in ship components and systems^19^. This approach allows for timely intervention, reducing maintenance costs and increasing equipment reliability. According to E. Ingemarsdotter et al.^20^, CBM implementation has significantly reduced unplanned downtime by up to 30% and maintenance costs by up to 25%. However, one of the challenges faced in the application of CBM is the requirement for reliable and accurate data collection systems. In 2021, R. Wang et al.^21^ deployed two distinct neural network models: one focused on evaluating the health and overall condition of the engine, while the other was geared towards predicting its operational functionality and susceptibility to breakdowns. To validate the efficacy of these models, operational datasets were gathered from real marine diesel engines operating under both fault conditions and normal circumstances. The findings underscored the potential of this framework to serve as an advanced online monitoring tool for prognosticating the performance of marine diesel engines.

Predictive analytics and ML algorithms have been increasingly utilized in the maritime industry to optimize maintenance decision-making. By analyzing historical data and identifying patterns, these methods can predict future failures, enabling ships to schedule maintenance activities based on data-driven predictions^22–24^. This approach has shown promising results by M. Payette and G. Abdul-Nour^25^ and P. L. Bokonda et al.^26^ with studies reporting a reduction in maintenance costs by up to 15% and an increase in equipment reliability by up to 24%. However, the effectiveness of predictive analytics and ML relies heavily on the quality and quantity of available data. Insufficient or inaccurate data can lead to unreliable predictions and suboptimal maintenance decisions. Remote monitoring and diagnostic systems have revolutionized maintenance practices in the maritime industry. By enabling real-time assessment of ship systems and components from onshore locations, these systems reduce the need for physical inspections and minimize down time. Through the use of sensors, connectivity, and advanced data analysis techniques, remote monitoring systems such as the internet of things (IoT), R. Krishnamurthi et al.^27^ have shown that maintenance costs decrease by up to 20% and equipment availability increases by up to 15%. However, it requires substantial investment in infrastructure and technology^28^.

Reliability-centred maintenance (RCM) is a strategy that prioritizes critical ship components and systems based on their importance and potential failure consequences, it optimizes maintenance efforts and ensures efficient allocation of resources. I. H. Afefy^29^ has shown that RCM implementation has led to a reduction in maintenance costs by up to 30% and a decrease in equipment failures by up to 25%. However, the successful implementation of RCM requires a comprehensive understanding of a ship’s systems and the potential consequences of failure. Additionally, the complexity of modern vessels poses challenges in identifying critical components and determining appropriate maintenance intervals^30^. Through the exploration of these methodologies, several problems have been either solved or partially solved. For instance, the implementation of CBM, predictive analytics, remote monitoring, and RCM has resulted in a substantial reduction in unplanned downtime, maintenance costs, and equipment failures^31^. By leveraging advanced data analysis techniques and real-time monitoring, these methods have improved the overall reliability, safety, and operational efficiency of marine vessels.

In a study conducted by S. Lion et al.^32^, a comprehensive thermodynamic model of a two-stroke, low-speed, 13.6 MW marine diesel engine was developed using the Ricardo WAVE engine simulation software. A low pressure (LP) exhaust gas recirculation (EGR) architecture was implemented to evaluate the engine’s performance in compliance with IMO tier III regulations. Findings indicate that the combined utilization of innovative emission reduction strategies and waste heat recovery (WHR) systems, such as organic Rankine cycle (ORC), enables the development of marine diesel engines that exhibit fuel consumption levels comparable to those achieved under tier II operation, while significantly reducing emissions. A preliminary economic analysis suggests potential annual fuel cost savings of approximately 5% when operating with ORC^33^. This research highlights the potential of these strategies to enhance engine performance and contribute to the overall optimization of marine diesel engines.

In 2020, V. Fernoaga et al.^34^ studied the optimization of engine emissions and performance which involved detailed examinations of exhaust system configurations where correlations among sensor-acquired engine data are increasingly assessed through AI methodologies. The impact of exhaust back pressure (EBP) on engine performance, particularly effective power, was investigated using a turbocharged diesel engine on a dynamometric test-bench. This study expanded upon conventional cloud/edge-computing models by integrating advanced AI algorithms and software frameworks.

According to recent research by Z. H. Munim et al.^35^, the field of maritime research has identified four prominent clusters of AI applications in relation to big data which encompass digital transformation, the utilization of big data from the automatic identification system (AIS), energy efficiency, and predictive analytics. Research within this domain focuses on various areas, including autonomous ships, big data analytics, AI algorithms, cybersecurity, IoT, and virtual reality. These advancements hold tremendous promise for revolutionizing the maritime industry. In addition, the concept of the internet of vessels (IoV) has been proposed as an innovative approach to maritime transport. The IoV represents a network of intelligent and interconnected vessels and shore facilities, incorporating various digital entities. It integrates key technologies such as sensing, automation, telecommunications, information systems, computers, and intelligent control into a unified platform. Research has indicated that the utilization of eco-ships with patented fuel-saving and emission-reduction systems, container technology, big data solutions for ship information management, and system automation are among the most effective strategies in this context^36^.

In 2014, R. Krakowski^37^ made functional systems out of a marine diesel engine piston, which were portrayed in terms of diagnostic testing. This breakdown made it possible to identify diagnostic systems, the most common failure sites, and those whose malfunction could have major consequences. Results have shown that most of the faults found in marine diesel engines are damage to the injection system. Computerization of measurement systems leads to their use with the simultaneous use of AI, especially in specialized systems.

Recently, A. Kiritsi et al.^38^ explored various types of liquefied natural gas (LNG) engines, fuel options, and modern propulsion systems and evaluated how AI techniques have been employed to assess the performance of LNG engines in marine applications. The study’s findings indicate that existing AI models can generate reliable predictions and be utilized to optimize propulsion parameters for LNG ships. In 2025, V. R. Menda et al.^39^ developed data-driven predictive models for a common rail direct injection (CRDI) diesel engine operating with advanced fuel blends using support vector regression (SVR), random forest (RF), and decision tree (DT) algorithms. The results showed that ensemble learning approaches, particularly RF, achieved superior prediction accuracy for key performance indicators (KPIs) as well as emission parameters^40,41^. Recently, D. P. Viana et al.^42^ focused on the application of multilayer perceptron (MLP) and RF neural networks to evaluate the absolute value of failure severity for diesel engine prognosis. A comprehensive database comprising 3500 failure scenarios was developed, and failures were categorized and quantified through the application of RF regression models and artificial neural networks. By implementing this advanced predictive maintenance framework, the study demonstrates a significant enhancement in the ability to predict and prevent diesel engine failures.

In 2025, S. Mylapalli et al.^43^ conducted an integrated experimental and ML-based analysis to evaluate the impact of Fe₃O₄ nanoparticle-enhanced Sterculia Foetida biodiesel blends on compression-ignition engine performance and emissions. The results demonstrated that ensemble-based ML models achieved very high predictive accuracy, R values exceeding 0.99, confirming their capability to capture nonlinear relationships between engine operating parameters and response variables^44^. C. Hickenbottom^45^ discussed the use of a condition indicator to detect sensor failures. The approach was also implemented on an oil pressure transducer (OPX) algorithm as applied to Honeywell turbofan engines. This algorithm focuses specifically on oil pressure monitoring and prediction to prevent engine failures related to oil pressure anomalies. This focused approach allows for more accurate and timely interventions, reducing the risk of unexpected engine failures and associated downtime.

While previous literature has explored the use of AI and ML algorithms for the diagnosis of various failures to start maintenance actions before issues escalate, the use of forecasting algorithms is yet to be employed for the prediction of failures especially in marine diesel engines, such proactive approach can protect engines from unexpected downtime and maintenance costs^46^. Therefore, the aim of this research is to develop and implement an innovative proactive maintenance framework specifically designed to enhance the reliability, longevity, and overall performance of marine diesel engines through the early detection and prevention of potential failures. This will be grounded in controlled experimental simulations aimed at replicating a wide array of failure scenarios commonly encountered in marine diesel engines. These simulations will provide controlled environments for monitoring and recording critical operational parameters, with a particular emphasis on observing temperature fluctuations, vibration patterns, and other key metrics that serve as reliable indicators of component failures.

Through the strategic deployment of sensors and data acquisition systems, coupled with the application of ML algorithms and predictive analytic techniques, the research aims to transform vast volumes of raw operational data into actionable insights. State of-the-art supervised learning algorithms will be employed to discern subtle deviations and patterns within the data that precede and predict potential failures. Concurrently, detection models will be utilized to identify anomalies indicative of abnormal engine behaviour, which may signify impending mechanical issues or performance degradation.

Unlike most existing studies that rely solely on historical operational data or a limited number of fault indicators, this research introduces a comprehensive and experimentally validated framework for proactive maintenance of marine diesel engines. The novel framework of the proposed approach comprises the use of controlled experimental fault simulation to generate labeled datasets representing realistic failure modes using a multi-sensor approach, the integration of a ConvLSTM2D-based multivariate time-series prediction model capable of capturing both temporal dependencies and cross-parameter correlations among various engine parameters (cylinder temperatures, cooling water inlet and outlet temperatures, and vibration along 3 axes), and the coupling of future-state prediction with an RF classification model to enable early identification of fault scenarios before critical thresholds are reached.

## Methodology

**Fig. 2:**
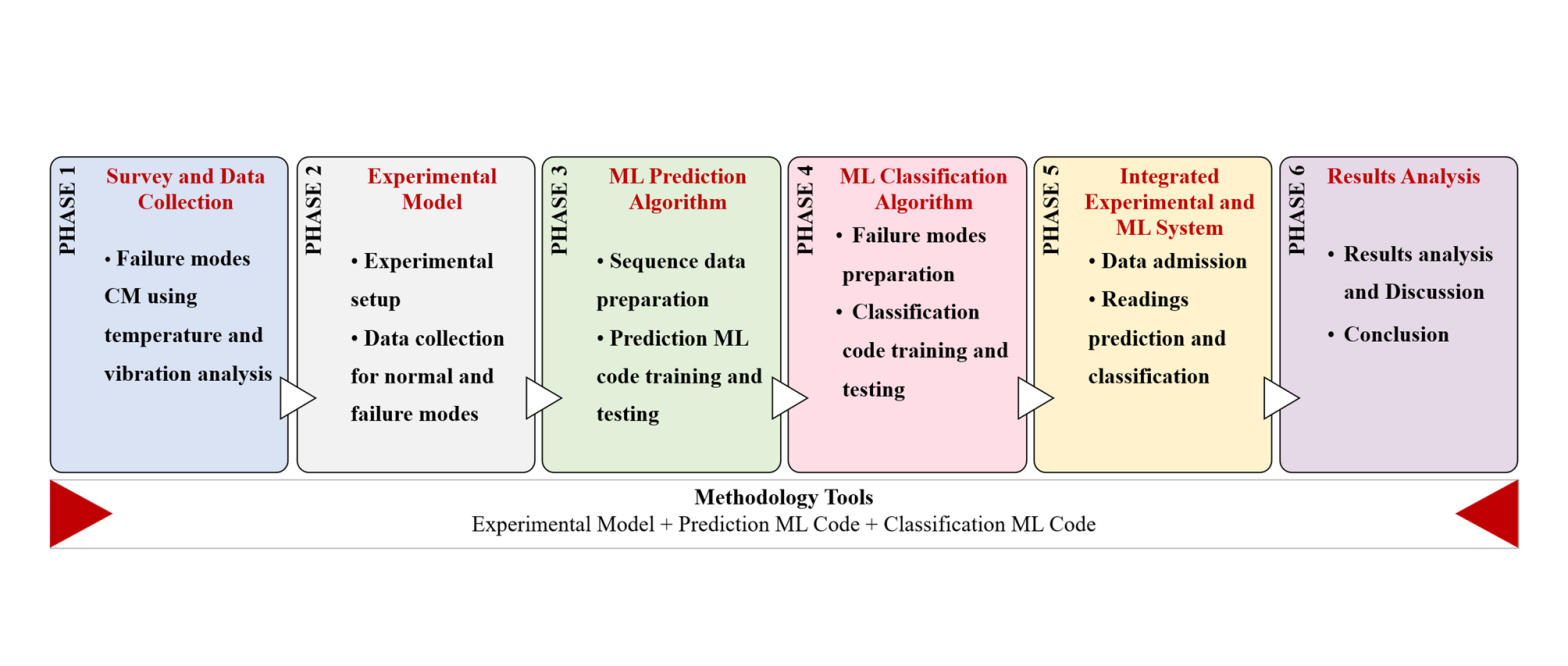
Overview of the proposed research methodology for marine diesel engines proactive condition monitoring and fault detection.

To address the problem statement, a comprehensive methodology is presented to integrate controlled experiments, advanced ML techniques, and systematic data analysis, as illustrated in Fig. 2. The primary objective of this methodology is to formulate a proactive maintenance strategy tailored for marine diesel engines. The experimental investigation is conducted using a 4-stroke, 4-cylinder diesel engine fitted with temperature sensors, tri-axial vibration sensors, and a tachometer system, enabling the acquisition of thermal, mechanical, and rotational data required for model development and validation.

A literature review was carried out to identify common failure modes in marine diesel engines and suitable implementation on the model engine^42^. Based on the review, a structured methodology was developed to support temperature- and vibration-based condition monitoring.

**Fig. 3:**
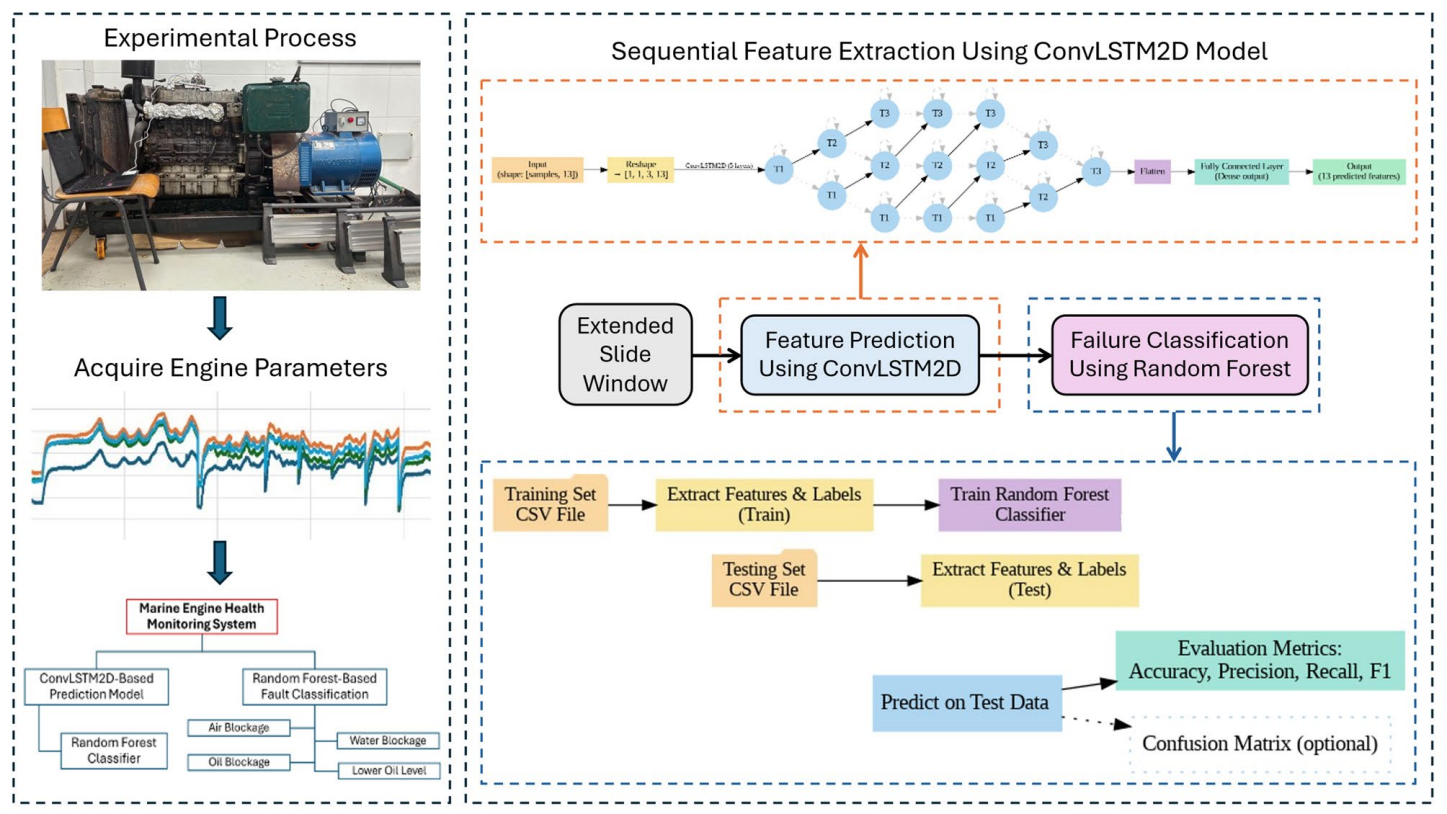
Detailed methodology of the proposed experimental and machine learning-based engine health monitoring framework.

Figure 3 illustrates the detailed methodology starting with the acquisition of different engine parameters from the experimental model under normal operation and failure modes. The dataset is then processed using a ConvLSTM2D-based prediction model to forecast sequential engine parameters, which are subsequently classified into specific failure modes using a Random Forest classifier. This integrated prediction–classification methodology forms the basis of the proposed engine health monitoring system.

### Experimental model

**Fig. 4:**
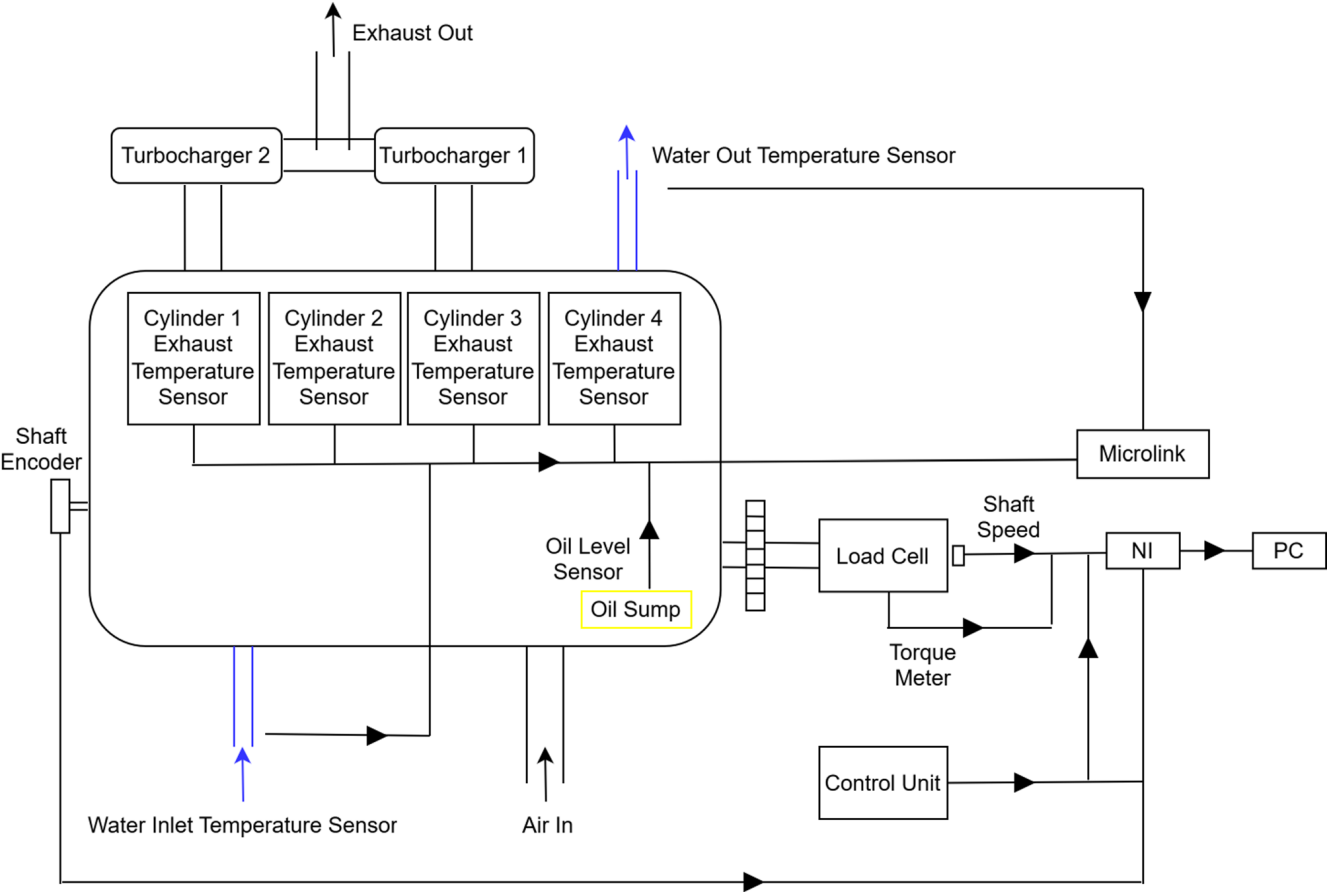
Layout and placement of condition monitoring (CM) sensors on the experimental diesel engine.

The experimental configuration was designed to simulate the effects of potential failures within the diesel engine and monitor the resulting variations in temperature and vibration. As shown in Fig. 4, sensors were cautiously chosen and strategically positioned throughout the engine. Temperature sensors were affixed to the exhaust pipe of each cylinder, while the vibration sensor was securely positioned on the engine block. The tacho system encompasses a tachometer connected to a transducer and mounted on the flywheel.

In the experimental setup, a Thermo King four-stroke, four-cylinder, water-cooled diesel engine was chosen for its reliability and performance. The engine’s principal technical specifications, as provided by the manufacturer, are summarized in Table 1.

**Table 1:** Manufacturer specifications of the Thermo King SE 2.2 diesel engine used in the experiments.

Specification	Value
Engine model	Thermo King SE 2.2 diesel engine
Type	Four-stroke, water-cooled
Number of cylinders	4
Cylinder arrangement	In-line vertical
Bore	3.465 in (88 mm)
Stroke	3.622 in (92 mm)
Displacement	136.6 in^3^ (2238.5 cm^3^)
Horsepower	34.8 hp @ 2200 rpm
Firing order	1-3-4-2
Compression ratio	20:1

**Fig. 5:**
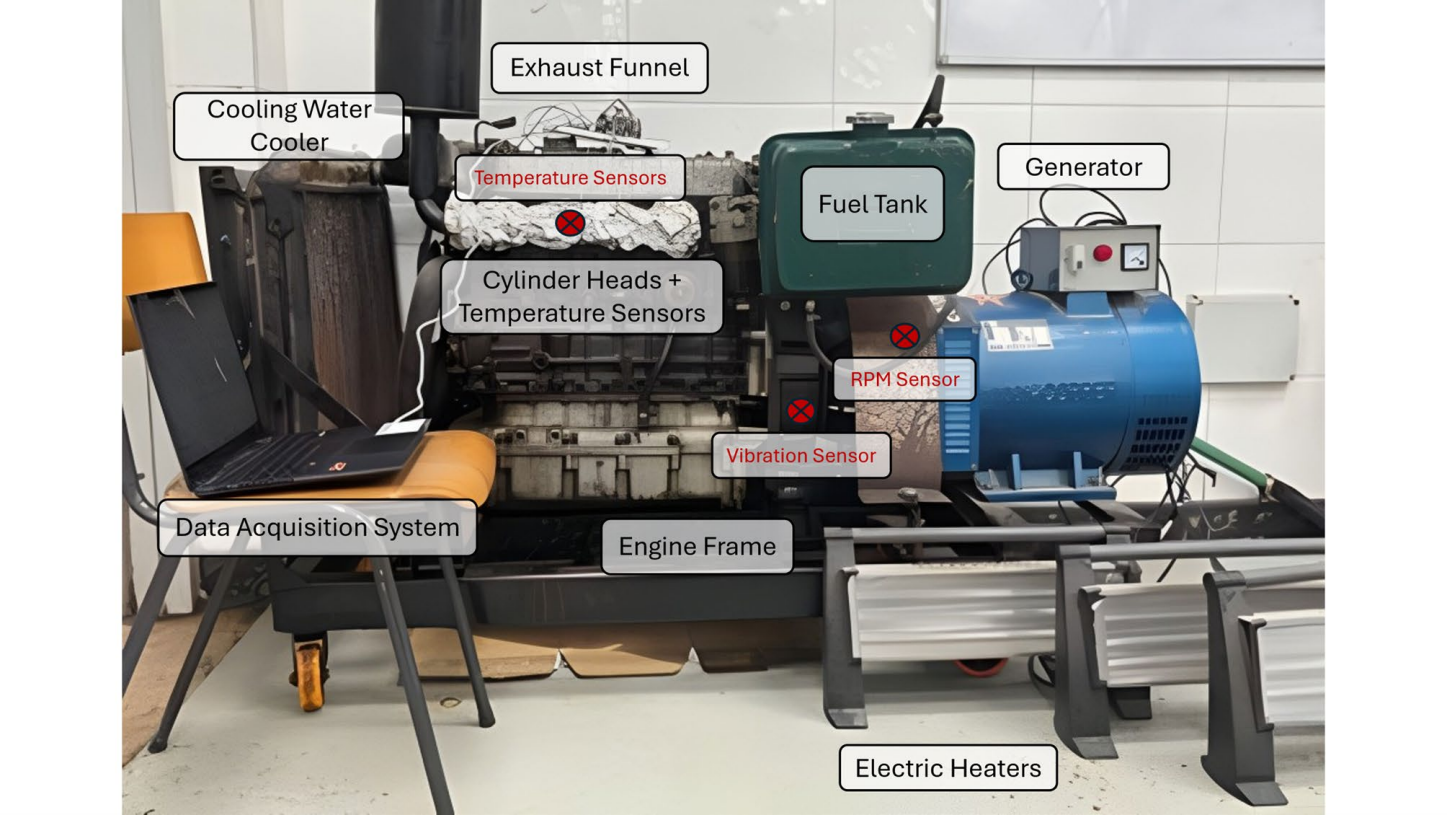
Experimental setup of the diesel engine test rig showing the placement of key subsystems and condition monitoring instrumentation.

The experimental investigations were conducted using the test rig shown in Fig. 5, which integrates the diesel engine, electrical loading system, cooling water and exhaust gas arrangements, and a centralized data acquisition unit. The engine was coupled to a generator to enable controlled load conditions, while dedicated temperature, vibration, and rotational speed sensors were installed to capture the engine’s thermal and mechanical responses under both normal operation and failure modes.

**Fig. 6:**
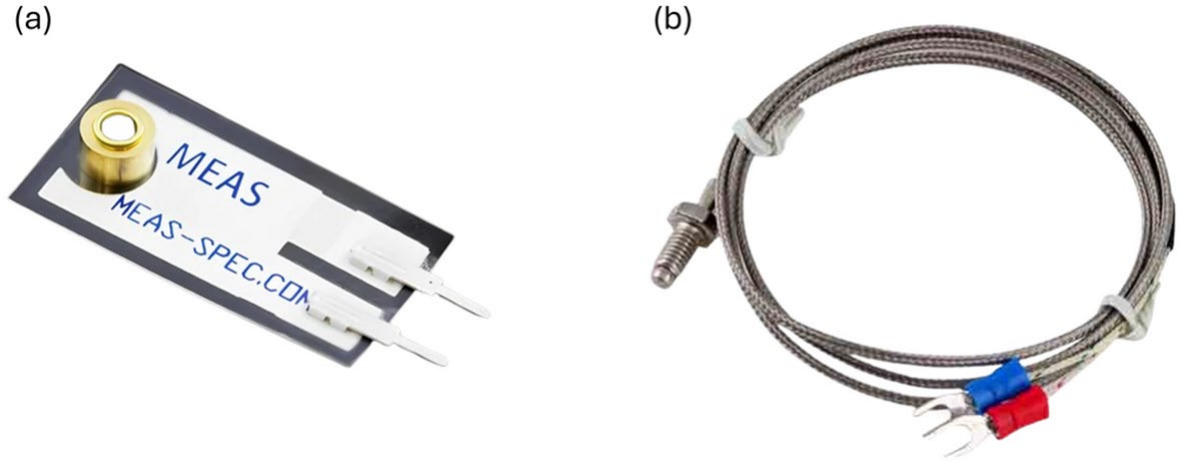
Experimental sensors used for engine data acquisition: (a) piezoelectric polymer (PVDF) film vibration sensor and (b) Type-K thermocouple temperature sensor.

The experimental data were acquired using a combination of thermal and vibration sensors, as illustrated in Fig. 6. A piezoelectric polymer (PVDF) film sensor was employed to capture engine vibration signatures. Temperature measurements were obtained using Type-K thermocouples installed at critical engine locations, including the exhaust manifold, cylinder heads, cooling water lines, and oil sump.

The vibration sensor was mounted on the engine block to capture vibration variations associated with mechanical wear and lubrication-related faults. To ensure measurement repeatability and consistency across all experimental runs, the mounting location was selected to be rigid, permanently accessible, and easily reproducible. Measurement uncertainty for vibration was evaluated by combining repeatability-based uncertainty (Type A), obtained from repeated steady-state measurements, with instrument-related uncertainty (Type B), derived from sensor specifications. For the triaxial piezoelectric polymer (PVDF) vibration sensor, the expanded uncertainty was estimated as ± 0.0395 m/sec^2^ (k ≈ 2) over the operating frequency range, which is adequate for resolving relative changes in vibration amplitude and patterns associated with abnormal engine behaviour.

Cylinder head temperature sensors were installed in close proximity to the exhaust ports of each cylinder to accurately capture combustion-related thermal variations. The exhaust manifold was tapped, and Type-K thermocouples were threaded directly into the manifold for each cylinder to ensure consistent thermal contact and repeatable positioning. Temperature measurement uncertainty was assessed by combining the standard uncertainty derived from repeated measurements (Type A) with the thermocouple tolerance specified by IEC standards (Type B). For the operating temperature range of the engine, the expanded uncertainty of the temperature measurements was estimated as ± 1.73 °C (k ≈ 2), with the overall uncertainty dominated by the thermocouple tolerance rather than measurement repeatability.

Cooling water temperatures were measured at both the inlet and outlet of the engine cooling cycle using Type-K thermocouples installed directly upstream of the engine block cooling channels. This placement ensured that the sensors were fully submerged in the cooling water flow, minimizing thermal lag and measurement bias.

In addition to thermal and vibration measurements, the electrical operating parameters of the engine, including voltage, current, and power, were continuously monitored, along with engine rotational speed expressed in revolutions per minute (RPM). Electrical measurements were acquired using calibrated instrumentation, with expanded uncertainties of approximately ± 1% for both voltage and current. Engine speed was measured using a tachometer–transducer system with an accuracy of ± 1 rpm. For derived quantities such as electrical power, uncertainty propagation was applied based on the uncertainties of the corresponding voltage and current measurements.

Sensor placement was determined based on engine geometry and operational characteristics. All sensors were mounted using appropriate mechanical fixtures to minimize vibration-induced noise and ensure stable thermal contact. Overall, the quantified measurement uncertainties and sensor placement strategy ensure that the acquired dataset reflects the thermal, mechanical, and electrical behaviour of the engine, thereby providing a robust foundation for the subsequent ML-based prediction and classification analysis.

This comprehensive dataset obtained was essential for understanding engine behaviour and facilitating early detection of potential failures. This approach ensures a rapid and accurate response to temperature changes. The collected readings underwent thorough processing using advanced ML techniques, leveraging Python code for modelling purposes. This analytical approach enhanced the ability to discern deviations in parameters resulting from failures or environmental variations, thereby safeguarding the overall performance and efficiency of the engine.

To effectively capture the dynamic behaviour of the engine and promptly identify potential failures, a suitable data collection frequency was established. This frequency was carefully determined by considering the engine’s operating conditions, including RPM and load, as well as the anticipated response time of components susceptible to potential failures. Preliminary tests were conducted to ascertain the optimal data collection frequency, ensuring an adequate number of data points while avoiding data overload. Temperature readings were systematically recorded at regular intervals, typically spanning from 30 to 60 sec, to provide a comprehensive understanding of thermal dynamics. Simultaneously, the vibration sensor continuously monitored levels of vibration throughout the engine’s operation.

An analysis of the engine’s structure was conducted to pinpoint key areas where temperature variations serve as indicative signals of potential failures. Building upon this analysis, the minimum number of sensors essential for precisely capturing the required data was determined. In the preliminary phases of the experiment, foundational assumptions were made to establish a baseline for subsequent comparisons. These assumptions were derived from historical data of the engine’s performance characteristics. The assumption readings served as a reference point for identifying deviations from anticipated behaviour and the timely detection of potential failures. Test scenarios were implemented to study various engine failure modes, such as air inlet duct blockage, exhaust gas duct blockage, cooling water inlet blockage, and low lubrication oil level.

To refine this baseline, historical maintenance records were leveraged to categorize the collected data into two distinctive instances: normal and failure cases. Instances corresponding to engine failures or situations requiring maintenance were explicitly labelled as one of the failure modes established. This categorization process enhanced the recognition of patterns associated with normal operation and deviations indicative of potential issues. Data acquisition systems, specifically tailored to accommodate the selected sensors, were deployed to acquire precise sensor readings. These systems captured, processed, and stored sensor data in real-time, forming a cohesive integration with the ongoing engine operations. The recorded data were systematically organized in a tabular format to facilitate clarity and accessibility.

**Fig. 7:**
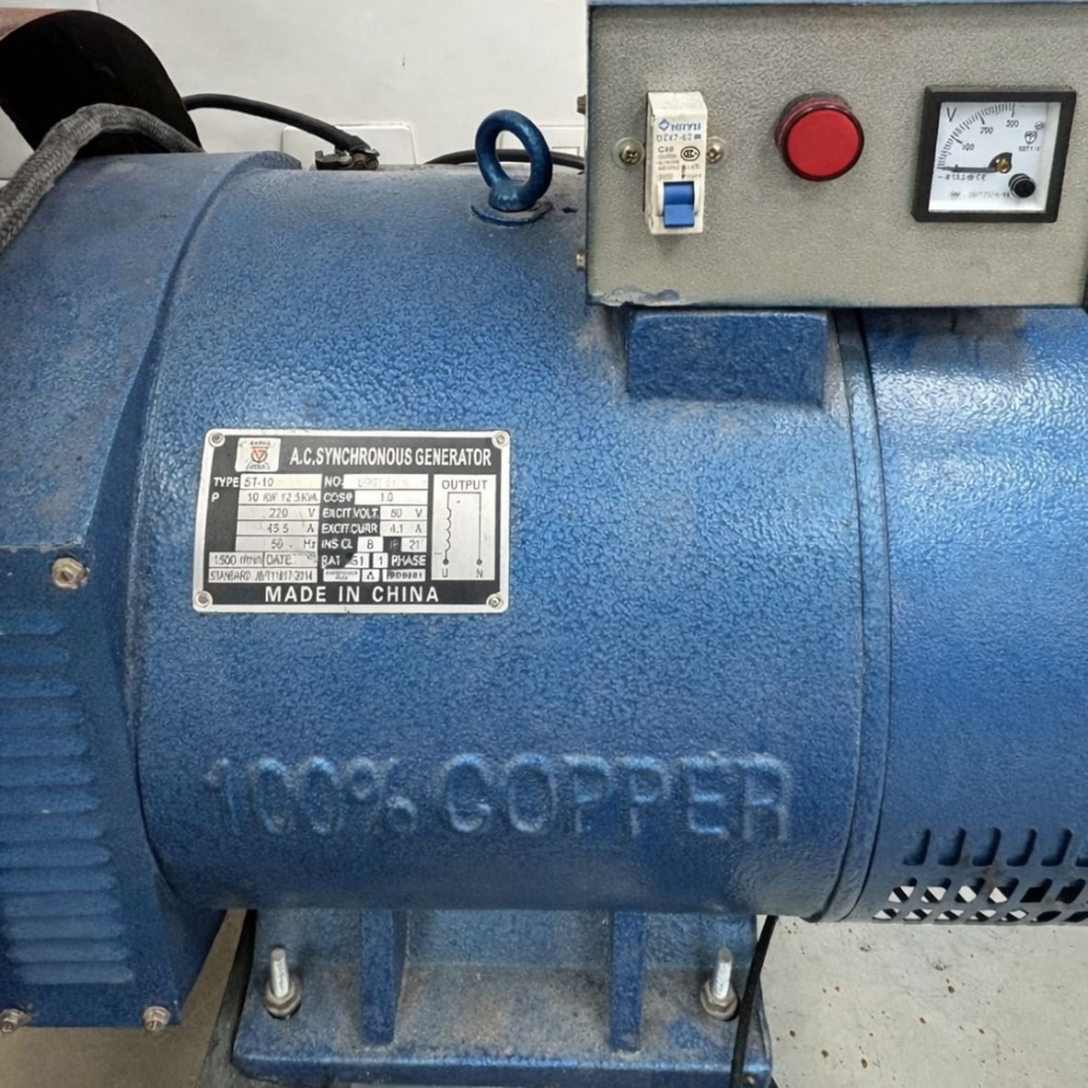
Single-phase 10 kW alternator used to apply controlled electrical load to the diesel engine during experimental testing.

The 10-kW alternator in Fig. 7 selected proved to be ideal for the experimental setup as it was able to load the engine effectively while accurately measuring current and voltage.

**Fig. 8:**
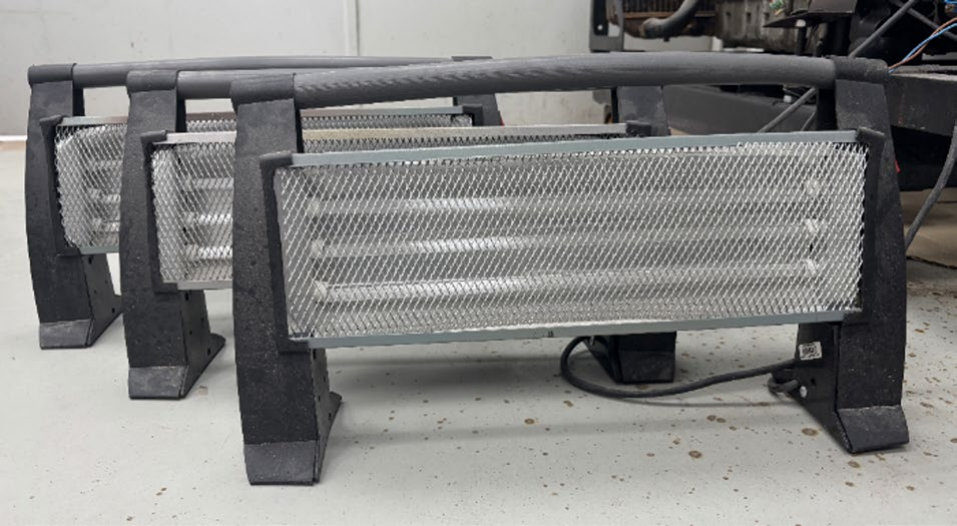
Resistive electrical load comprising three 1800 W heaters connected in parallel.

This setup included integrating three 1800 W heaters, shown in Fig. 8, through 6 mm wires in parallel to ensure efficient power distribution and reliable performance across different operational scenarios. This alternator is tailored for single-phase applications, operating frequencies of 50–60 Hz, operational speeds of either 1500 rpm or 1800 rpm, and voltage ranging from 220 V to 240 V.

In this study, five distinct failure modes were carefully implemented to evaluate the performance and response of the system under various operational conditions. The first mode represented normal operation without any faults, serving as a baseline for comparison and establishing standard operational metrics. The second mode examined air inlet blockages, implemented in three distinct stages: 25%, 50%, and 75% blockage. This allowed for a thorough understanding of how increasing levels of blockage affect system performance. The third failure mode focused on exhaust outlet blockages, also divided into three stages: 25%, 50%, and 75% blockage. This mode was designed to mimic the impact of exhaust the impact of exhaust blockage on the system. In the fourth failure mode, water inlet blockages were tested at two levels: 25% and 50%. This was aimed at identifying how reduced water flow influences the cooling system’s efficiency and overall engine performance. The fifth failure mode investigated the effects of low oil levels. Tests were conducted at two critical points: a low oil level (25%) and a critically low oil level (50%). This mode was particularly important for understanding the potential risks and performance degradation associated with inadequate lubrication. Each stage within these operational/failure modes was executed with precision over a 40-minute period. The procedure for each stage was as follows; for the first 10 min, the system was heated up to reach optimal operational temperature, ensuring consistent starting conditions. For the next 10 min, the system operated with no load, simulating normal operation and establishing a baseline for no-load performance, the following 10 min, a load of 5400 W was applied using electric heaters. This phase tested the system’s response under significant operational stress, and in the final 10 min, the system was allowed to cool down with no load, providing data on recovery and cooling efficiency. Throughout each stage, a comprehensive set of parameters was continuously monitored to gather detailed performance data. These parameters included the temperature of the four cylinder heads, providing insights into combustion efficiency and exhaust gas behaviour, temperature of the cooling water inlet and outlet, crucial for assessing the cooling system’s performance, vibrations in the x-, y-, and z-axes, which indicate mechanical stability and potential wear or faults, RPM which is essential for understanding engine speed and operational consistency, and voltage and current measurements which reflect the electrical load and generator performance.

By carefully implementing and monitoring these scenarios, the study aimed to provide a thorough understanding of how various faults and operational conditions affect the system’s performance, reliability, and safety.

### ML prediction algorithm

**Fig. 9:**
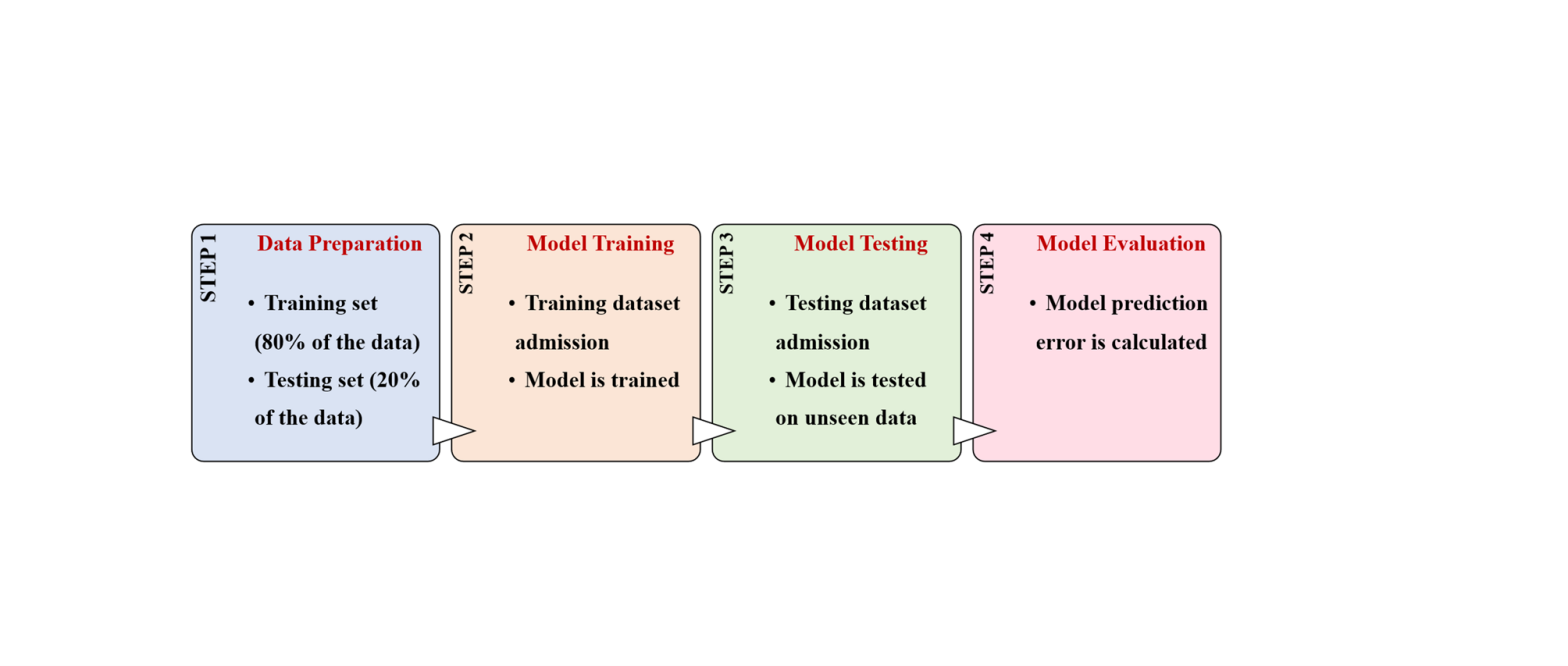
ML prediction methodology illustrating the sequential workflow adopted.

Regarding the data prediction methodology, a specialized algorithm designed to improve accuracy in forecasting trends was utilized as illustrated in Fig. 9. Central to the approach was the implementation of a long short-term memory (LSTM) model, specifically tailored for analysing sequential data in time series applications. This LSTM model offers two primary types: univariate and multivariate LSTM models. The univariate LSTM model focuses on predicting trends within a single variable over time, while the multivariate LSTM model expands this capability to predict multiple interrelated features simultaneously. This flexibility is particularly advantageous in scenarios where insights from diverse data points contribute to a comprehensive understanding and prediction of complex patterns and interactions.

The methodology employed in this study involves several key steps to utilize the ConvLSTM2D model effectively starting with obtaining an 80 − 20% portioning scheme for each failure mode^47^. A portion of 80% of the readings for each mode was used to train the ML model for the prediction of future readings. The remaining 20% was used to test the model. The training dataset in comma-separated values (CSV) format, containing multivariate time series data was loaded using Pandas. The dataset typically includes variables such as cylinder readings, water levels, RPM, voltage, current, power, and vibration measurements. Similarly, the test dataset is loaded to evaluate the model’s predictive performance on unseen data. Feature columns relevant to the forecasting task are identified and extracted from the dataset. These features were titled “Cylinder 1”, “Cylinder 2”, “Cylinder 3”, “Cylinder 4”, “Water Outlet”, “Water Inlet”, “RPM”, “Voltage”, “Current”, “Power”, “Vibration X”, “Vibration Y”, and “Vibration Z”. The ‘split sequences’ function was employed to divide the multivariate time series data into input-output pairs. This function creates sequences of specified length ‘n steps’ from the training data to facilitate sequential learning by the model and uses a sliding window approach which enables the model to frame the engine parameter data into fixed-length sequences to ensure that each training sample captures the dynamic behaviour of the system over time. The input data ‘X’ is reshaped to fit the input requirements of the ConvLSTM2D model.

**Fig. 10:**
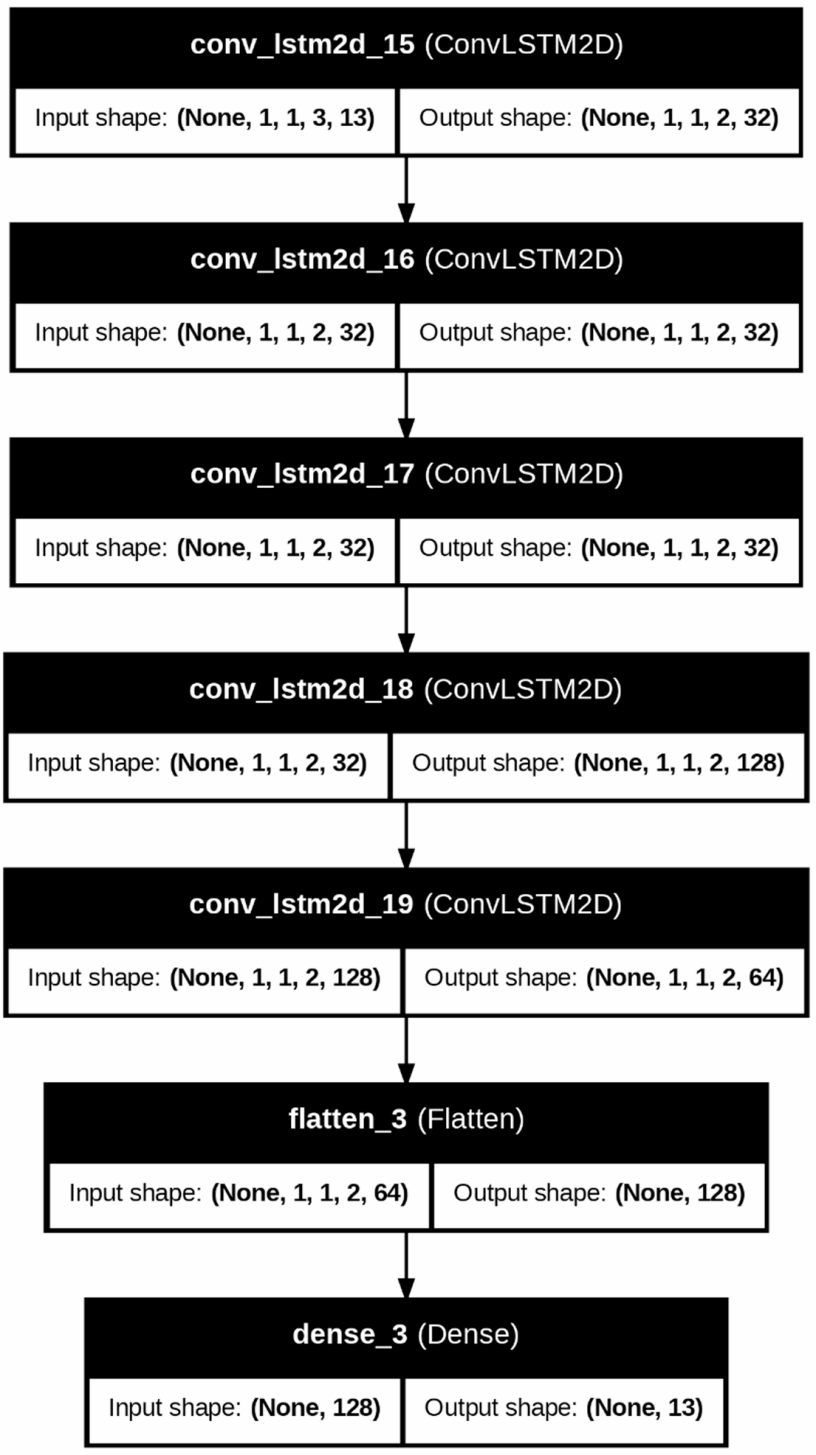
Architecture of the proposed ConvLSTM2D-based prediction model used for multivariate time-series forecasting of engine parameters.

The ConvLSTM2D model was defined using Keras Sequential API. This model architecture integrates convolutional layers with LSTM units, allowing it to learn spatial as well as temporal patterns within the data. The model was compiled with the Adam optimizer and the MSE loss function for training. The model was trained using the prepared input sequences ‘X’ and the corresponding output sequences ‘y’ over a specified number of epochs to optimize its predictive performance. The test data ‘x input values’ were reshaped similarly to the training data to ensure compatibility with the model’s input shape and predictions ‘yhat’ were generated using the trained ConvLSTM2D model on the reshaped test data ‘x input’. This step provided forecasted values for the multivariate time series variables. Considerations for model tuning, including iterative adjustments to ‘n steps’, filter numbers, kernel sizes, and optimization strategies, enhanced forecasting accuracy. The proposed model layers are displayed in Fig. 10. The best layers and parameters for this model were obtained through GridSearch.

The ConvLSTM2D prediction model was trained using a sliding-window sequence formulation with n_steps = 3 time steps and 13 input features (Cylinder 1–4 temperatures, cooling water inlet/outlet temperatures, RPM, voltage, current, power, and vibration X/Y/Z). Input sequences were reshaped to (samples, 1, 1, 3, 13) to match the ConvLSTM2D input format. The network consisted of five ConvLSTM2D layers followed by a Flatten layer and a Dense output layer producing a 13-dimensional forecast vector. The model was optimized using the Adagrad optimizer with MSE loss, trained for 200 epochs with a batch size of 64.

Evaluation metrics such as mean squared error (MSE) in Eq. (1), mean absolute error (MAE) in Eq. (2), and root mean squared error (RMSE) in Eq. (3) were used to gauge the model’s accuracy against actual data^48^.

1 $$\begin{array}{*{20}{c}} {MSE=\frac{1}{n}\mathop \sum \limits_{{i=1}}^{n} {{\left( {{Y_i} - \widehat {{{Y_i}}}} \right)}^2}} \end{array}$$

2 $$\begin{array}{*{20}{c}} {MAE=\frac{{\mathop \sum \nolimits_{{i=1}}^{n} \left| {{y_i} - {x_i}} \right|}}{n}} \end{array}$$

3 $$\begin{array}{*{20}{c}} {RMSE=\sqrt {\frac{{\mathop \sum \nolimits_{{i=1}}^{N} {{\left( {{x_i} - \widehat {{{x_i}}}} \right)}^2}}}{N}} } \end{array}$$

where, MSE is the mean squared error, n is the number of data points, Y_i_ is the observed value, $\widehat {{{Y_i}}}$ is the predicted value, MAE is the mean absolute error, y_i_ is the predicted value, x_i_ is the true value, RMSE is the root mean squared error, N is the number of non-missing data points, and $\widehat {{{x_i}}}$ is the estimated time series.

To train and test the model, an 80%-20% partitioning scheme was used for each dataset ensuring equal representation of each operation and failure modes in the training and testing sets while maintaining the time-series nature of the dataset. Error metrics were calculated for the developed ConvLSTM2D model.

### ML classification algorithm

**Fig. 11:**
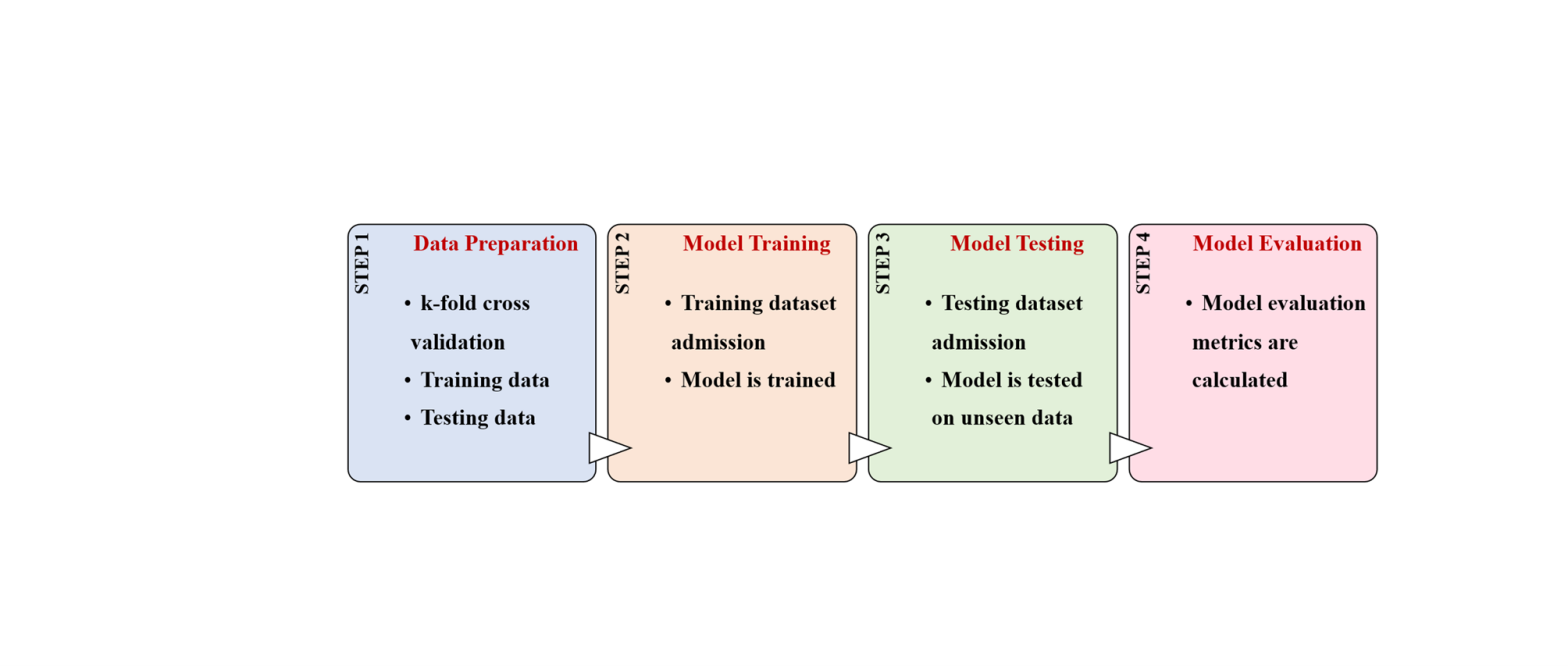
Machine-learning classification methodology adopted for failure mode identification.

An ML algorithm was developed to use the recorded engine parameters along with their failure modes to train and test various models; Logistic Regression, Random Forest (RF), Support Vector Machine (SVM), K-Nearest Neighbour (KNN), Naive Bayes, Decision Tree, Multi-Layer Perceptron (MLP), and AdaBoost to determine the highest accuracy model to be used to classify the forecasted data obtained from the ML prediction code to predict the failure modes the engine might encounter during operation as shown in Fig. 11. Data classification was then done using the RF model as it had the highest accuracy.

By using the scenario data and implementing the code, k-fold cross-validation was employed during the training phase. This technique involves partitioning the training dataset into k equal subsets or “folds.” The model is trained k times, each time using a different fold as the validation set and the remaining k-1 folds as the training set. The classification code uses an RF classifier to predict and classify the operational state of the engine. The classification aims to identify different fault scenarios by analysing temperature, vibration, and electrical parameters. The code was initialized by importing the necessary libraries for data manipulation, ML, and performance evaluation. Then the training dataset was prepared and loaded for the model.

The dataset contained features and labels representing different fault scenarios and was organized into features (inputs) and labels (outputs). The data was then split into training and testing sets to evaluate the model’s performance. An RF model was run on the training set then used to make predictions on the test set to evaluate its performance. Metrics such as accuracy, precision, and recall were calculated to assess the model’s performance. A confusion matrix was generated and visualized to show the classification results. Finally, the trained model was used to classify new data. This code provided a comprehensive approach to training the RF classifier, evaluating its performance, and using it for proactive maintenance of a diesel engine by identifying different fault scenarios based on engine parameters collected using various sensors.

The failure classification model employed an RF classifier trained using the same 13 input features used in the prediction stage (Cylinder 1–4 temperatures, cooling water inlet/outlet temperatures, RPM, voltage, current, power, and vibration X/Y/Z). The RF model was implemented using the Random Forest Classifier function in scikit-learn with default hyperparameters and trained on the classification training dataset. Model performance was evaluated using a separate testing dataset and reported using accuracy, macro-averaged precision, macro-averaged recall, macro-averaged F1-score, and the confusion matrix.

The evaluation matrix was calculated for each ML model investigated using the following equations to assess the effectiveness of each model^48^. Equation (4) was used to calculate the accuracy of each model defining the closeness of the generated results to the actual experimental results.

4 $$\begin{array}{*{20}{c}} {Accuracy=\frac{{TP+TN}}{{TP+TN+FP+FN}}} \end{array}$$

Precision describes how close various results of the same quantity are to one another and was calculated for each model using Eq. (5).

5 $$\begin{array}{*{20}{c}} {Precision=\frac{{TP}}{{TP+FP}}} \end{array}$$

Each model’s recall, which describes the cases the model predicted correctly, was obtained using Eq. (6).

6 $$\begin{array}{*{20}{c}} {Recall=\frac{{TP}}{{TP+FN}}} \end{array}$$

The f1-score of each model, which summarizes the precision and recall parameters by obtaining the harmonic mean, was calculated using Eq. (7).

7 $$\begin{array}{*{20}{c}} {f1 - score=2 \times \frac{{precision \times recall}}{{precision+recall}}} \end{array}$$

where TP indicates the true positive, TN; the true negative, FP; the false positive, and FN; the false negative.

### Integrated experimental and ML system

In this phase, a complete integration between the time series forecasting model and the failure mode classification model was demonstrated. This approach resembles a real-time implementation of the developed proactive maintenance system where future engine parameter behaviour is not only predicted but also categorized into failure scenarios.

**Fig. 12:**
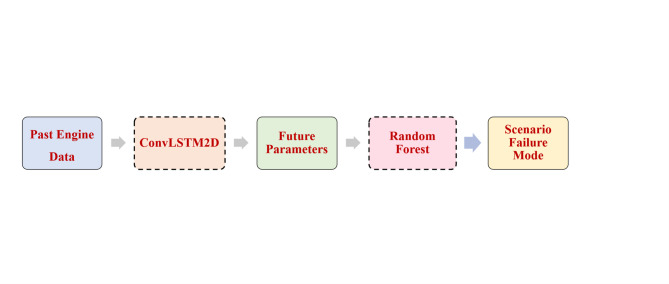
Implementation workflow of the proposed machine-learning framework.

The trained ConvLSTM2D model was used to predict future readings of the engine’s 13 features based on snippets of the testing dataset to ensure that the model works on real unseen data. The predicted features were then fed into the trained RF classifier to determine the failure mode of the engine. Figure 12 summarizes the integration process.

## Results and discussion

The results include the experimental data obtained from the diesel engine under normal operating conditions and multiple failure modes and their influence on the selected thermal, vibrational, and electrical parameters. Furthermore, the performance of the proposed ML algorithms was evaluated in comparison with other models used in time series forecasting and classification to assess the ConvLSTM2D model’s multivariate prediction under varying operational conditions, and the RF classifier’s accuracy in distinguishing fault modes based on the predicted engine parameters. Collectively, the results demonstrate how the combined prediction–classification approach supports robust proactive maintenance to prevent failures.

### Engine data acquisition

The operational / failure mode parameters were recorded and compared highlighting the effect of the failure modes on normal operational conditions of the engine. The collected dataset contained 21,454 records across all modes.

The engine operating parameters under both normal operation and imposed failure modes were recorded and analysed to evaluate the parameters’ deviation from baseline data based on each failure mode. The resulting dataset comprised 21,454 time series records including all fault categories and levels.

#### Air blockage failure mode

**Fig. 13:**
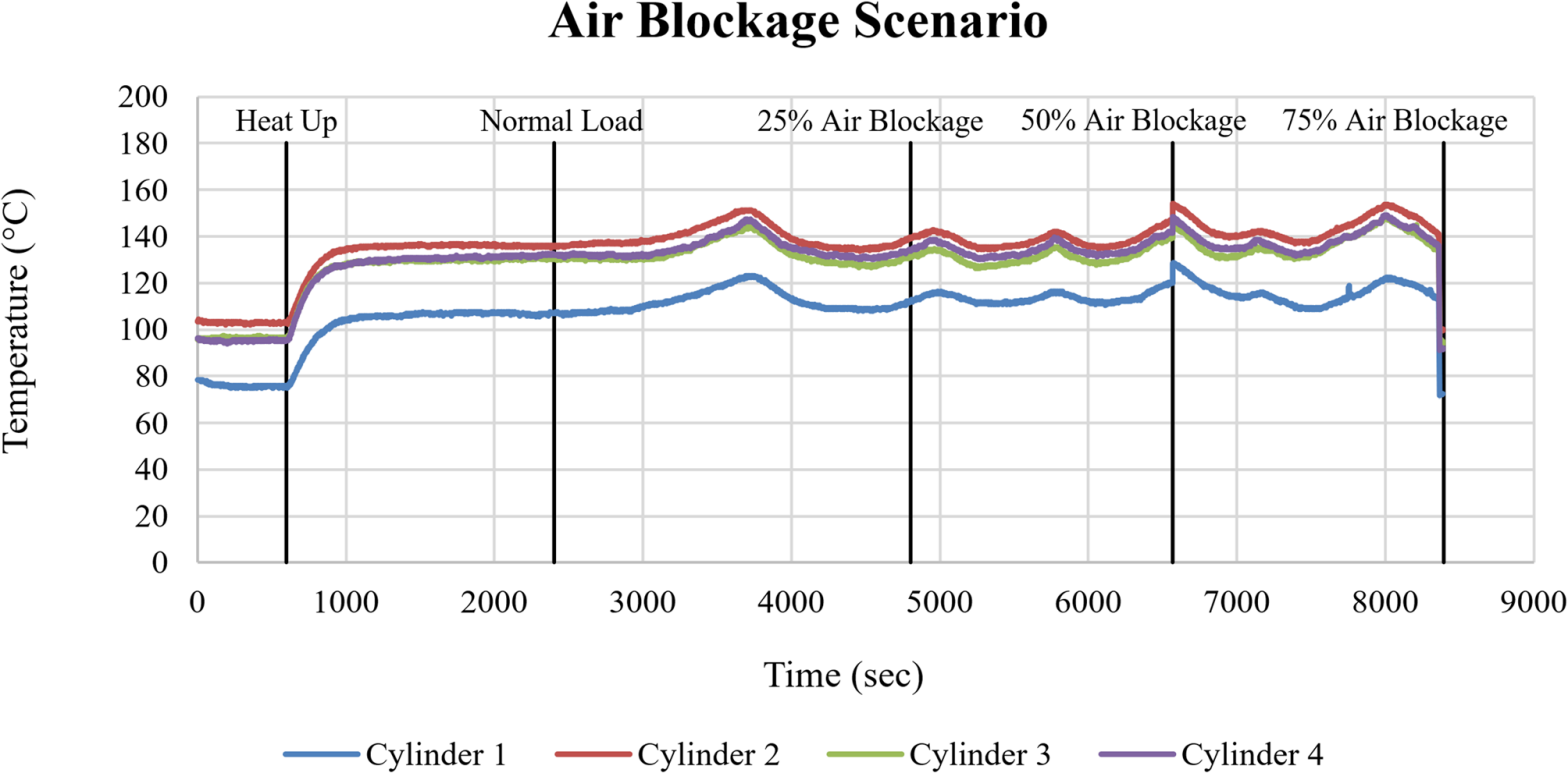
Cylinder head temperature response of Cylinders 1–4 (°C) under air blockage conditions as a function of time (sec).

Figure 13 illustrates the trend of cylinder head temperatures for Cylinders 1–4 under progressively imposed air blockage conditions. Following the initial heat-up phase, all cylinders reach steady operating temperatures after approximately 1000 sec, establishing a stable thermal baseline across all failure modes. Under normal load conditions (1000–3000 sec), Cylinders 2, 3, and 4 operate within a narrow temperature band with an average value of approximately 135 °C, while Cylinder 1 consistently stabilizes at a lower temperature of approximately 110 °C across all failure modes.

As air blockage was introduced, an increase in cylinder head temperature was observed across all cylinders. Relative to normal operation, the mean temperature rise was approximately ΔT ≈ 3–5 °C at 25% blockage, ΔT ≈ 10–13 °C at 50% blockage, and ΔT ≈ 15–18 °C at 75% blockage. Throughout all blockage levels, Cylinder 2 remained the hottest, while Cylinder 1 remained the coolest. At higher blockage severities (50% and 75%), short-term temperature oscillations increased, with peak-to-peak variations rising from approximately ± 2 °C under normal load to ± 4–5 °C at 75% blockage.

The gradual increase in cylinder head temperatures with increasing air blockage reflects the progressive reduction in excess air availability, which reduces combustion efficiency and increases thermal loading. The consistent cylinder temperature (Cylinder 2 highest, Cylinder 1 lowest) across all blockage levels indicates that this distribution is a result of engine geometry and cooling characteristics rather than by the blockage itself.

**Fig. 14:**
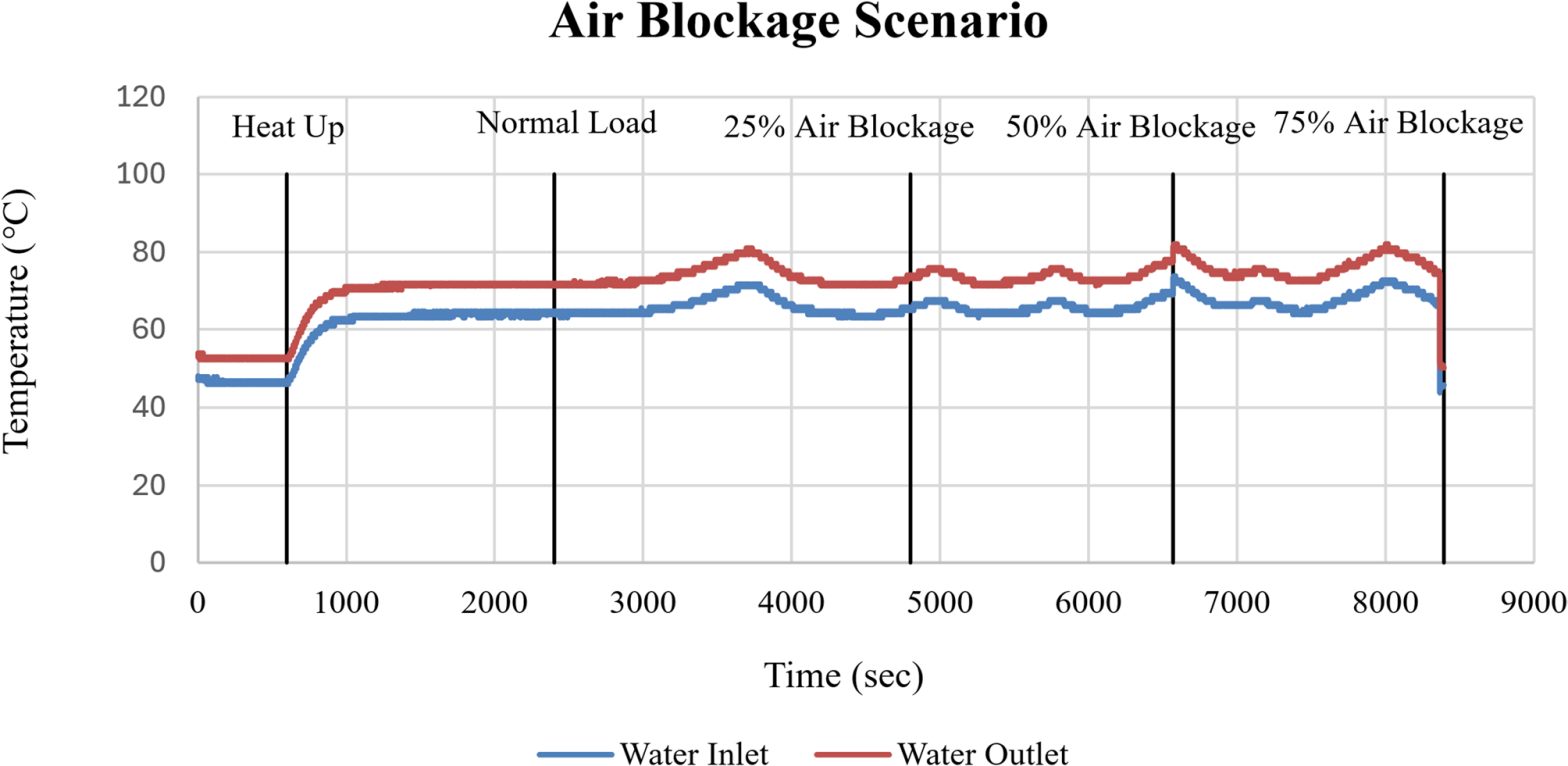
Cooling water inlet and outlet temperatures (°C) as a function of time (sec) under air blockage conditions.

The thermal response of the cooling system under air blockage conditions is shown in Fig. 14 through the cooling water inlet and outlet temperatures. Across all operating scenarios, the cooling water inlet temperature remained relatively stable, indicating consistent coolant supply conditions independent of air blockage severity. In contrast, the cooling water outlet temperature increased progressively with increasing air blockage. Relative to normal operation, the outlet temperature exhibited a clear upward shift at 25%, 50%, and 75% blockage, reflecting reduced heat rejection effectiveness as air intake becomes increasingly restricted.

The rise in cooling water outlet temperature, despite a stable inlet temperature, confirms that air blockage indirectly impacts the engine’s cooling effectiveness. Reduced air availability leads to higher combustion temperatures and increases heat transfer to the cooling system, resulting in elevated outlet temperatures. The increased temperature changes observed at higher blockage levels are attributed to combustion instability caused by insufficient air–fuel mixing.

#### Oil blockage failure mode

**Fig. 15:**
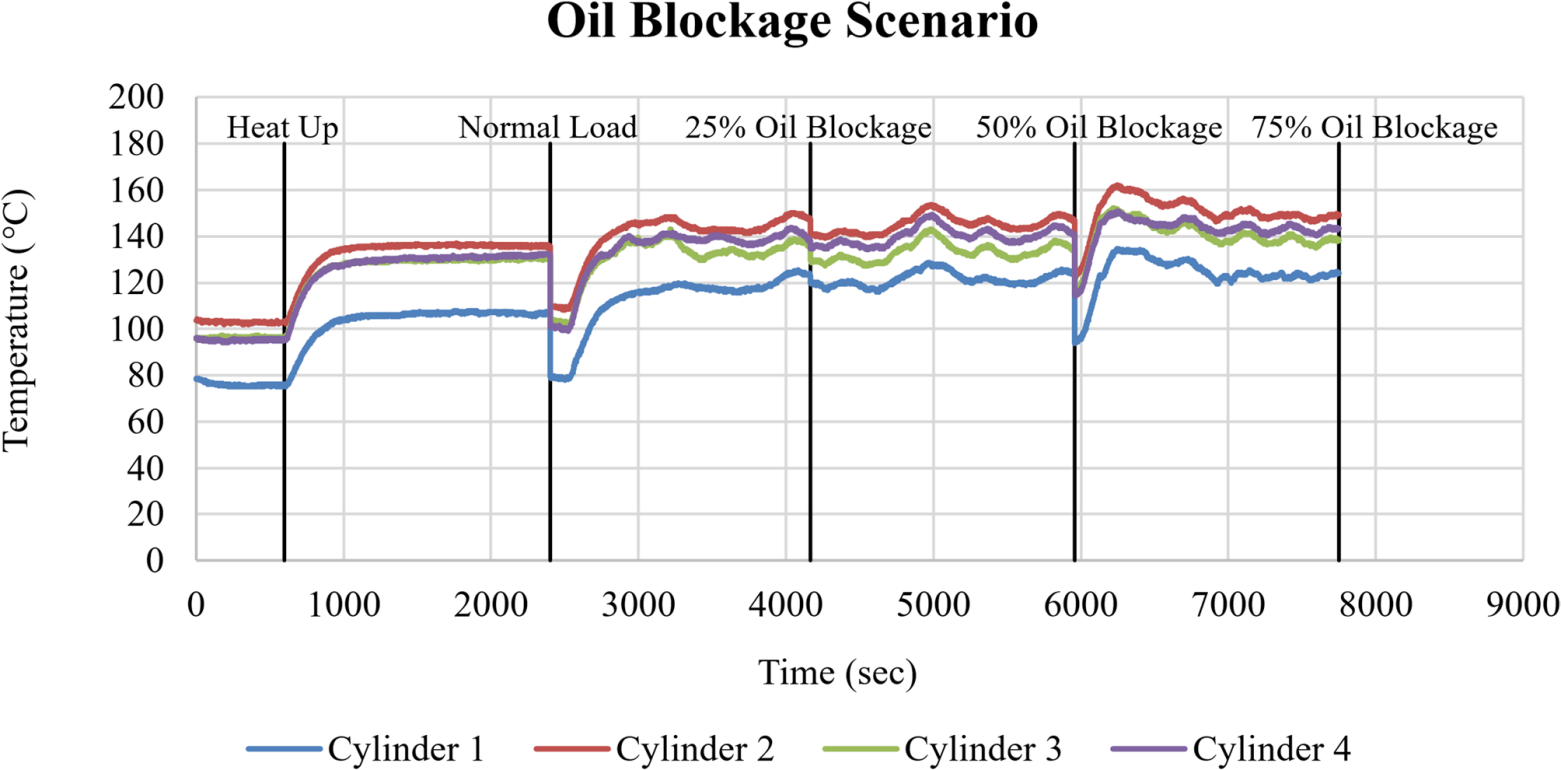
Cylinder head temperature response of Cylinders 1–4 (°C) under oil blockage conditions as a function of time (sec).

Figure 15 presents the rise of cylinder head temperatures for Cylinders 1–4 under progressively imposed lubricating oil blockage conditions.

The introduction of 25% oil blockage (at approximately 4000 sec) resulted in a slight increase in cylinder head temperatures across all cylinders. Relative to normal operation, the mean temperature rise was approximately ΔT ≈ 8–10 °C, with Cylinder 2 reaching peak values close to 150 °C following a short transient. At 50% blockage (initiated at approximately 6000 sec), a further increase in temperature was observed, with mean values rising by ΔT ≈ 18–20 °C compared to baseline conditions. Although transient fluctuations were present, the system settles into a higher thermal equilibrium.

The 75% oil blockage condition (starting at approximately 7600 sec) produced the most severe thermal response, with peak cylinder head temperatures approaching 155–160 °C.

The increase in cylinder head temperature with increasing oil blockage reflects the combined effects of reduced lubrication effectiveness and reduced auxiliary heat removal as oil restriction increases frictional heat generation.

**Fig. 16:**
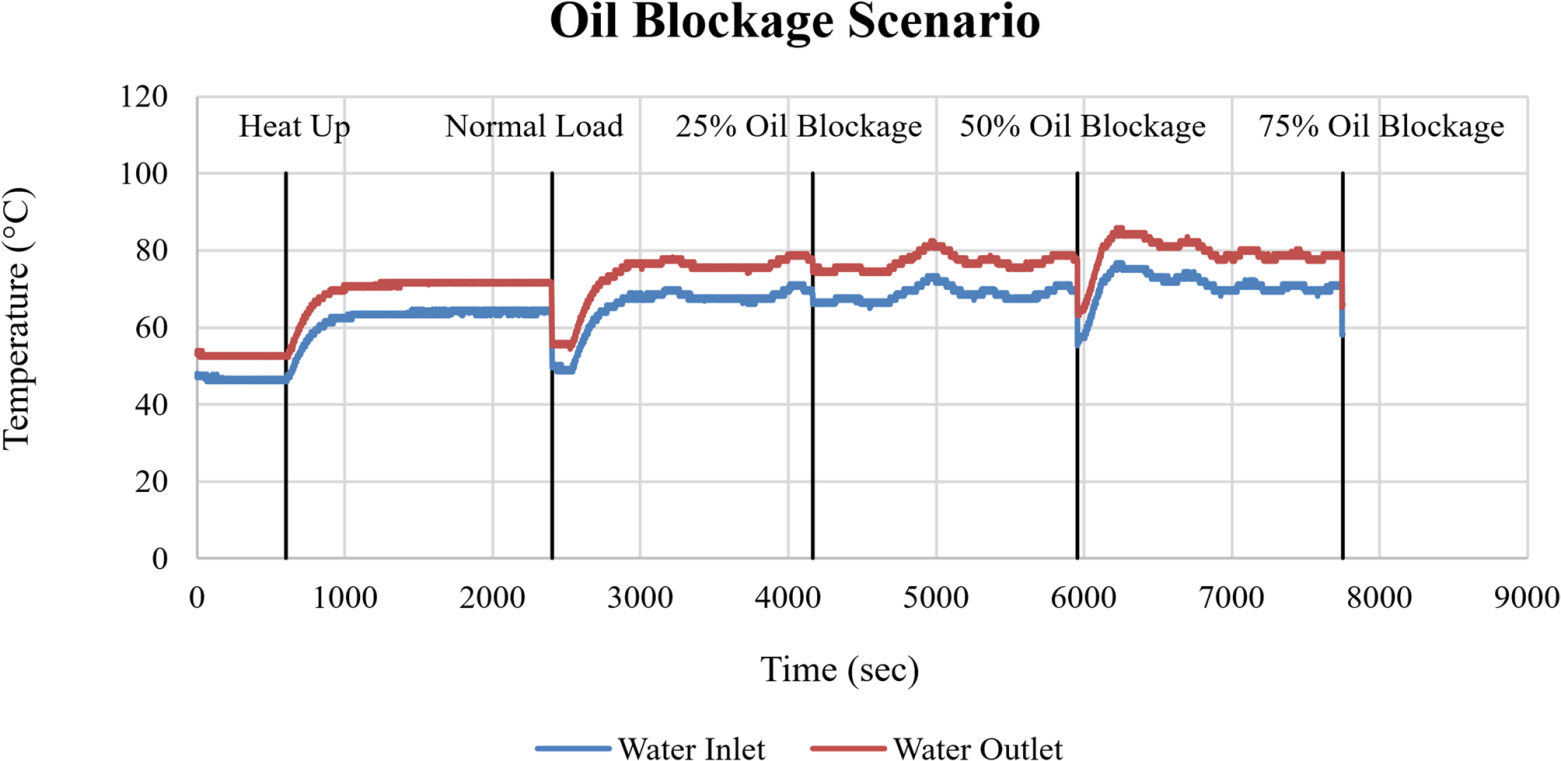
Cooling water inlet and outlet temperatures (°C) as a function of time (sec) under oil blockage conditions.

The corresponding cooling system response is shown in Fig. 16 through the cooling water inlet and outlet temperatures. The cooling water outlet temperature increased progressively with increasing oil blockage, indicating increasing thermal load transferred from the engine to the cooling circuit.

The progressive rise in cooling water outlet temperature confirms that oil blockage indirectly affects overall cooling performance by increasing the thermal energy that must be dissipated by the cooling water.

#### Cooling water blockage failure mode

**Fig. 17:**
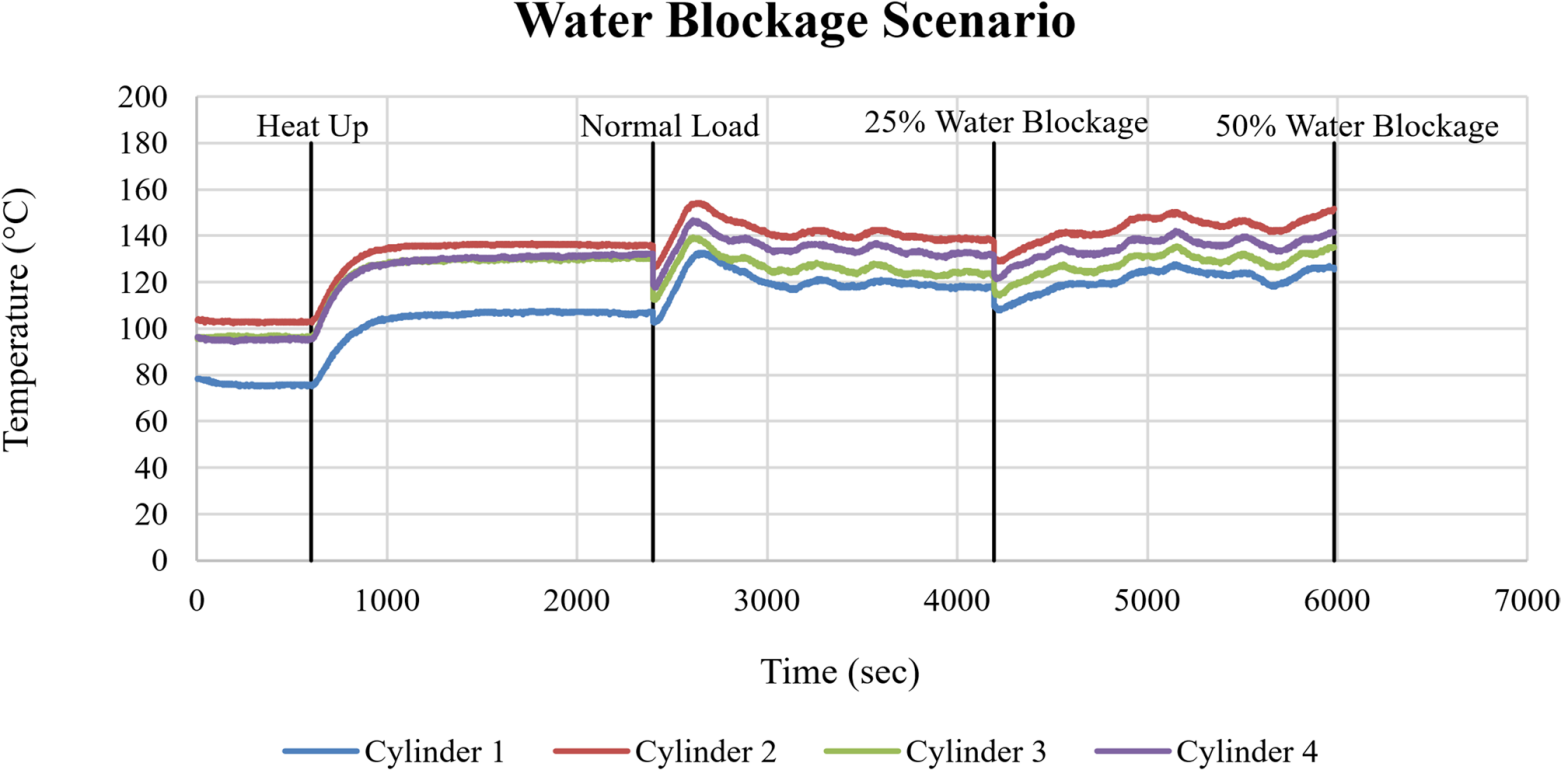
Cylinder head temperature response of Cylinders 1–4 (°C) under water blockage conditions as a function of time (sec).

Figure 17 illustrates the cylinder head temperatures for Cylinders 1–4 under progressively imposed cooling water blockage conditions. The introduction of 25% cooling water blockage (at approximately 4000 sec) produced an immediate and pronounced temperature increase across all cylinders. Relative to normal operation, peak cylinder head temperatures increased by approximately ΔT ≈ 12–15 °C, resulting in sharp transient spikes followed by partial stabilization at an elevated thermal baseline. At 50% blockage (initiated at approximately 6000 sec), the thermal response intensified further, with peak temperatures rising by approximately ΔT ≈ 18–20 °C compared to normal load conditions.

The sudden temperature spikes observed immediately after the introduction of cooling water blockage reflect the direct reduction in the engine’s heat rejection capacity. Cooling water restriction limits convective heat transfer almost instantaneously, leading to rapid accumulation of thermal energy within the cylinder heads.

**Fig. 18:**
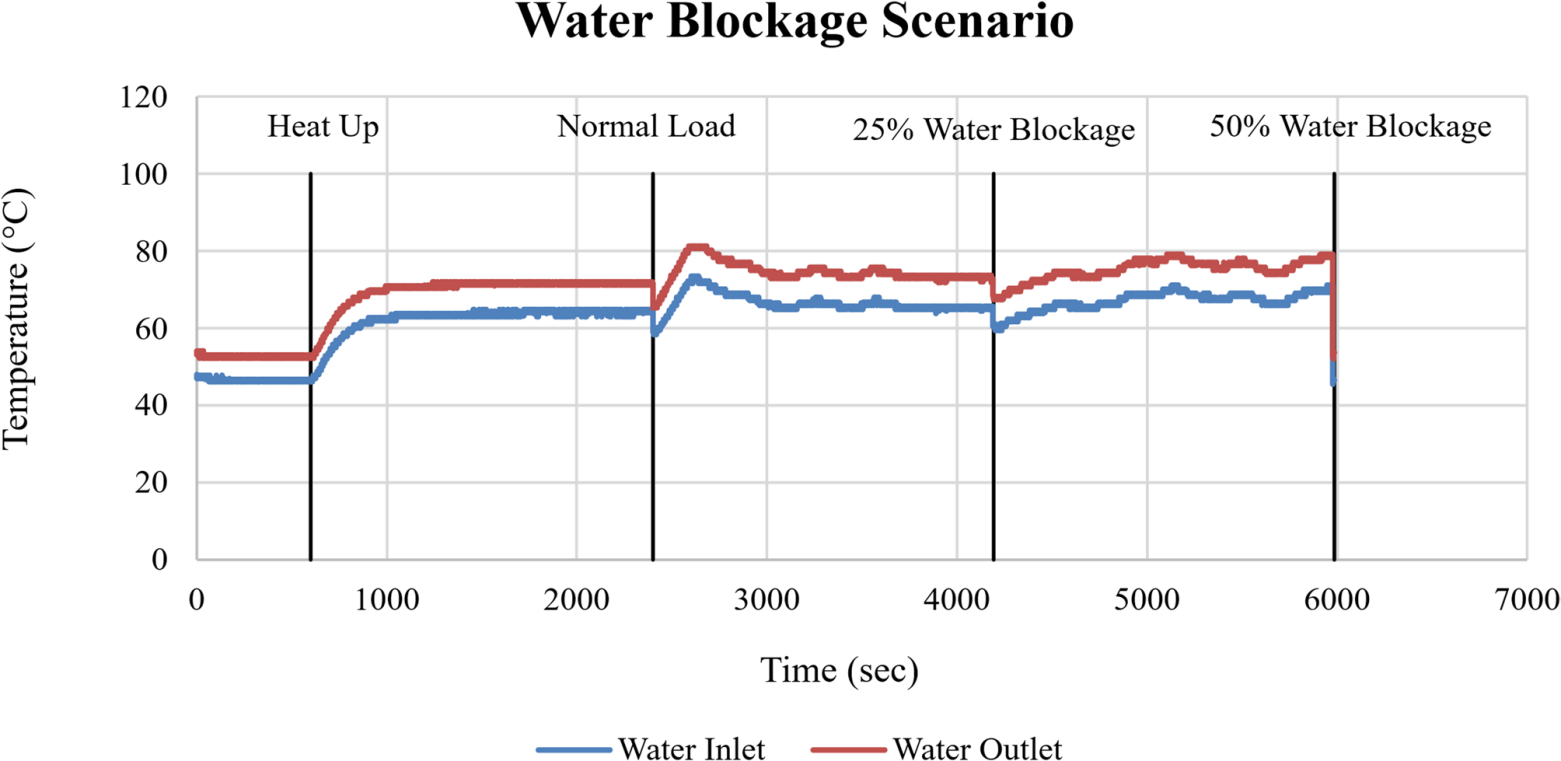
Cooling water inlet and outlet temperatures (°C) as a function of time (sec) under water blockage conditions.

The corresponding response of the cooling system is shown in Fig. 18 through the cooling water inlet and outlet temperatures. Relative to normal operation, outlet temperatures exhibited clear upward shifts at 25% and 50% blockage, reflecting reduced heat removal capability as coolant flow becomes increasingly restricted.

The rise in cooling water outlet temperature confirms that the cooling cycle is subjected to increased thermal loading under blockage conditions. The partial stabilization following the initial spikes indicates limited short-term thermal adaptation; however, the elevated temperatures demonstrate thermal stress.

#### Lower oil level failure mode

**Fig. 19:**
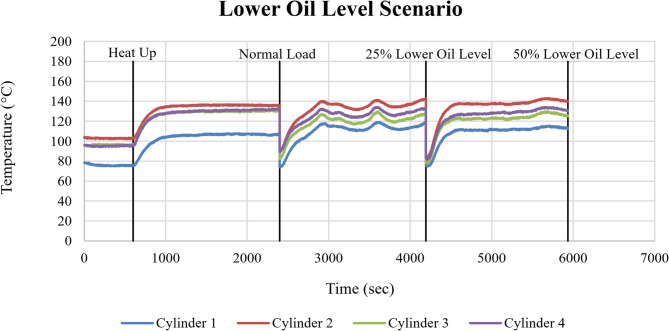
Cylinder head temperature response of Cylinders 1–4 (°C) under lower oil level conditions as a function of time (sec).

Figure 19 illustrates the trend of cylinder head temperatures for Cylinders 1–4 under progressively reduced lubricating oil level conditions. A reduction in lubricating oil level by 25% produced a consistent increase in cylinder head temperatures across all cylinders. Relative to normal operation, the mean temperature rise was approximately ΔT ≈ 4–6 °C, indicating a mild degradation in lubrication effectiveness. When the oil level was reduced by 50%, the thermal response became significantly more pronounced, with mean cylinder head temperatures increasing by approximately ΔT ≈ 7–10 °C compared to baseline conditions.

The observed temperature increase with decreasing oil level reflects the direct relationship between lubrication availability and frictional heat generation within the engine. At moderate oil reduction (25%), lubrication remains partially effective. However, at 50% oil level reduction, lubrication becomes insufficient to sustain effective hydrodynamic separation between moving components, leading to increased frictional losses and elevated cylinder head temperatures.

**Fig. 20:**
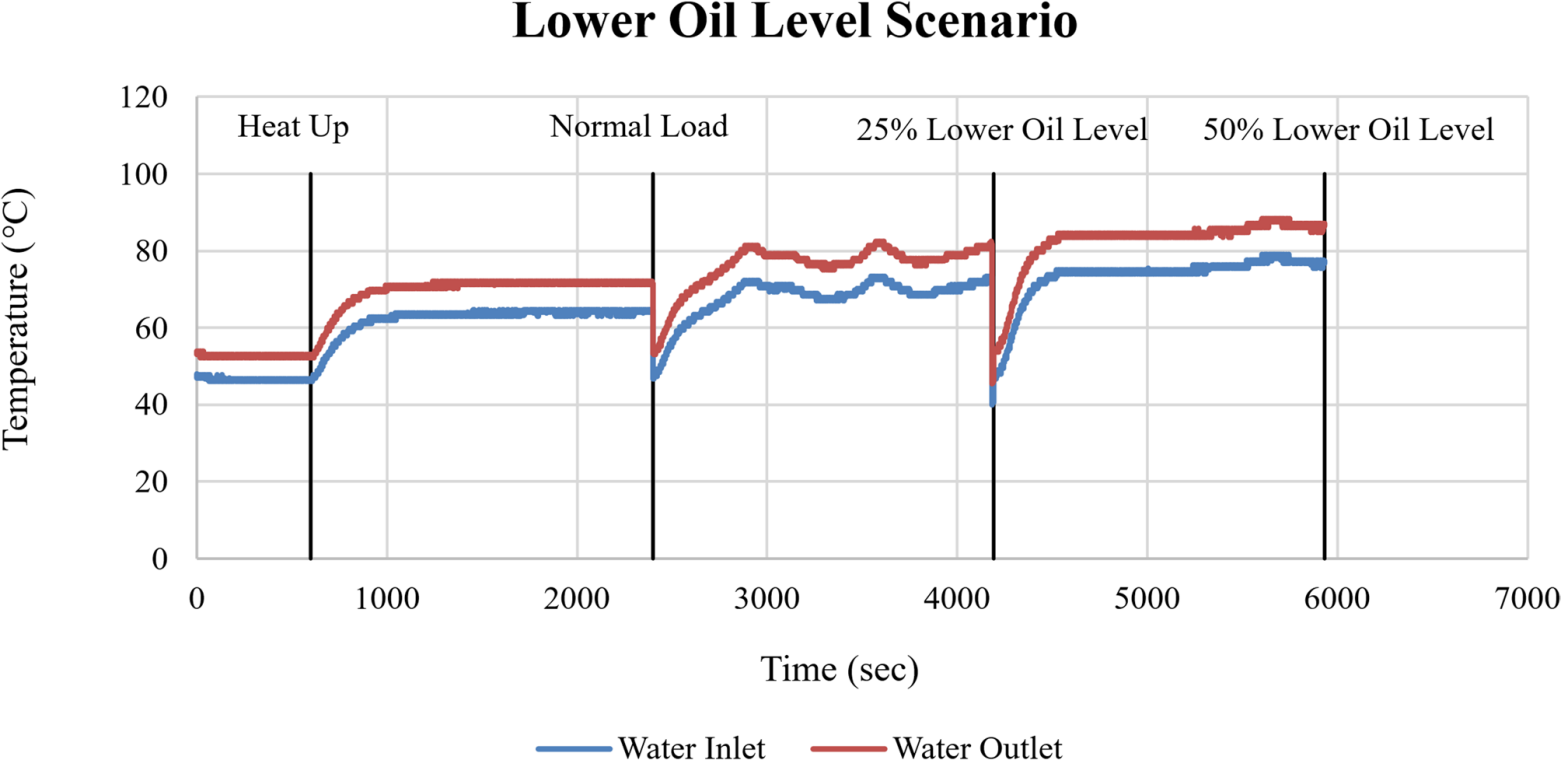
Cooling water inlet and outlet temperatures (°C) as a function of time (sec) under lower oil level conditions.

The corresponding cooling system response is shown in Fig. 20 through the cooling water inlet and outlet temperatures. Relative to normal operation, outlet temperatures exhibited clear upward shifts at both 25% and 50% oil level reduction, indicating increased thermal energy transfer from the engine to the cooling circuit.

The rise in cooling water outlet temperature confirms that reduced lubrication indirectly affects overall thermal management by increasing the heat that must be dissipated by the cooling system. The lower oil level fault appears as a gradual thermal stress, driven by friction-induced heat generation.

### Engine features prediction

**Fig. 21:**
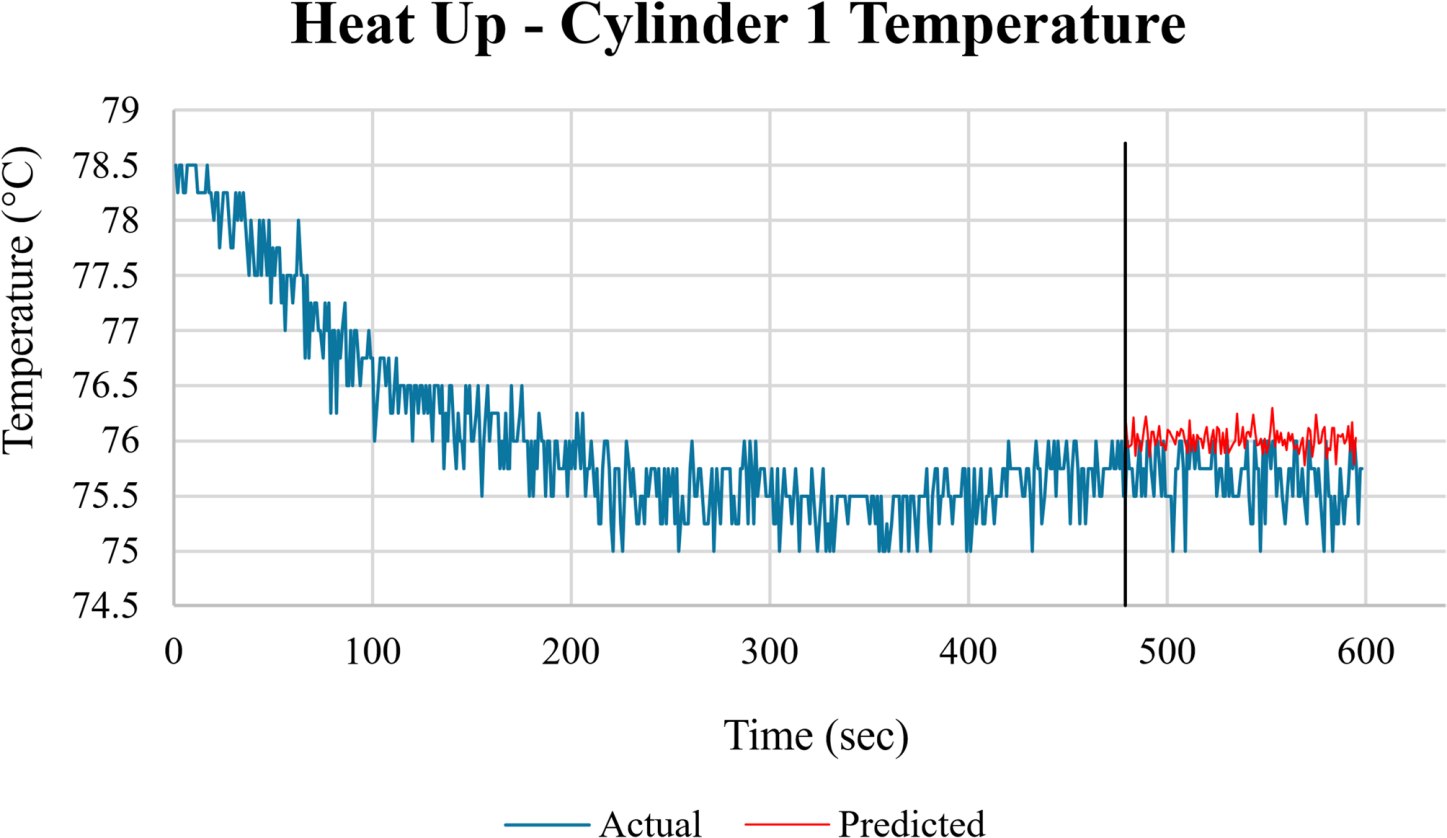
Comparison between actual and ConvLSTM2D-predicted cylinder head temperature of Cylinder 1 (°C) during the heat-up phase as a function of time (sec).

After implementing the code, each engine parameter was predicted along the test duration. The predicted data revealed consistent readings across multiple parameters, underscoring stable mechanical conditions during the measurement period. The model’s prediction results showed the actual and predicted data along with the error margin in each case for selected engine parameters, such as RPM, power, and vibration levels. These features were chosen based on their prediction errors (MSE) to visually emphasize the model’s performance. The graph shown in Fig. 21, using the train and test data, compares the actual temperature values with the predicted temperature values for cylinder 1 during the heat-up mode.

The actual temperature values started at around 78 °C and decreased steadily until about the 500-second mark, where they stabilized around 75.5 °C with some fluctuations. The predicted temperature values began after the 500-second mark, showing a relatively stable temperature around 75.5 °C with minor fluctuations. The ML model’s predictions closely followed the trend of the actual temperature values. This indicates that the model is effective in capturing the temperature dynamics during the heat-up phase.

**Fig. 22:**
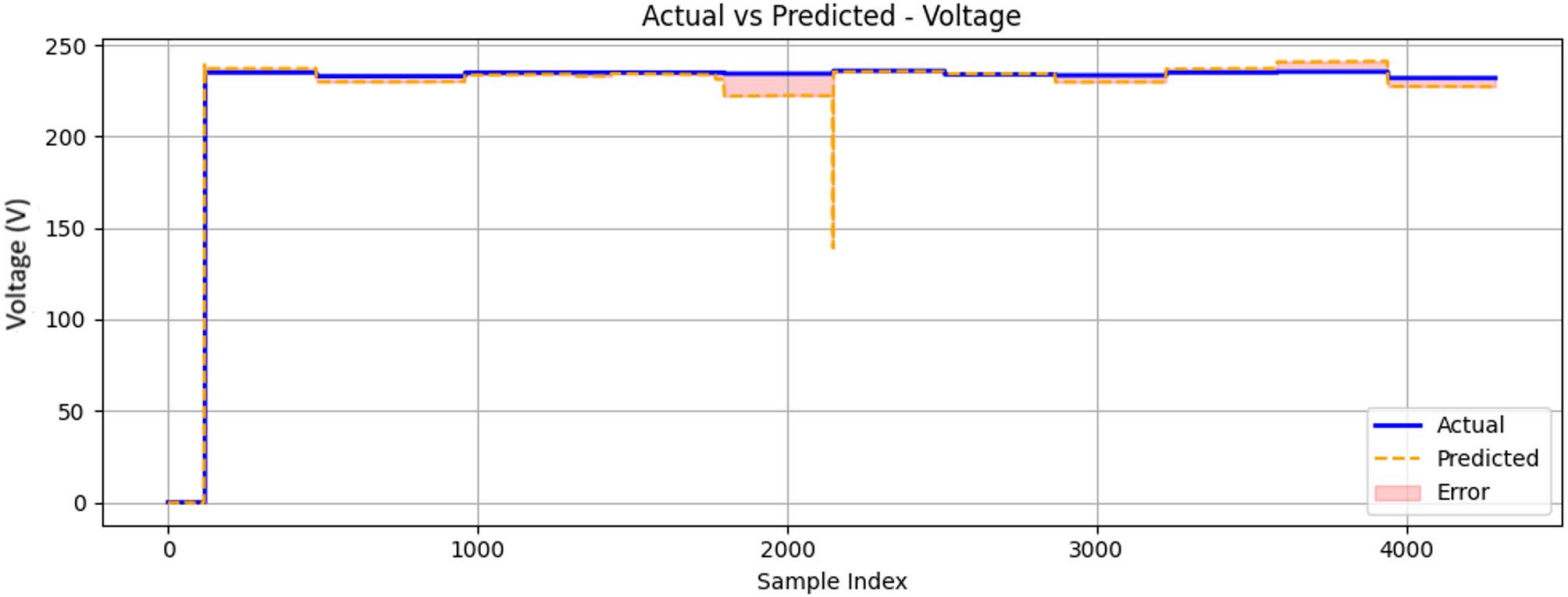
Comparison between measured and ConvLSTM2D-predicted generator output voltage (V) as a function of sample index during engine operation.

Figure 22 compares the voltage reading using the test data and those predicted by the model throughout the test duration. The algorithm maintained a strong correlation with experimental measurements across the operational range (2000–4000 sample index). Predicted values tracked the actual voltage curve with remarkable consistency, particularly in the mid-range (sample index of 3000) operating conditions where the error margin narrowed significantly. This suggests the model has effectively learned the electrical system’s characteristic behaviour during normal engine operation.

**Fig. 23:**
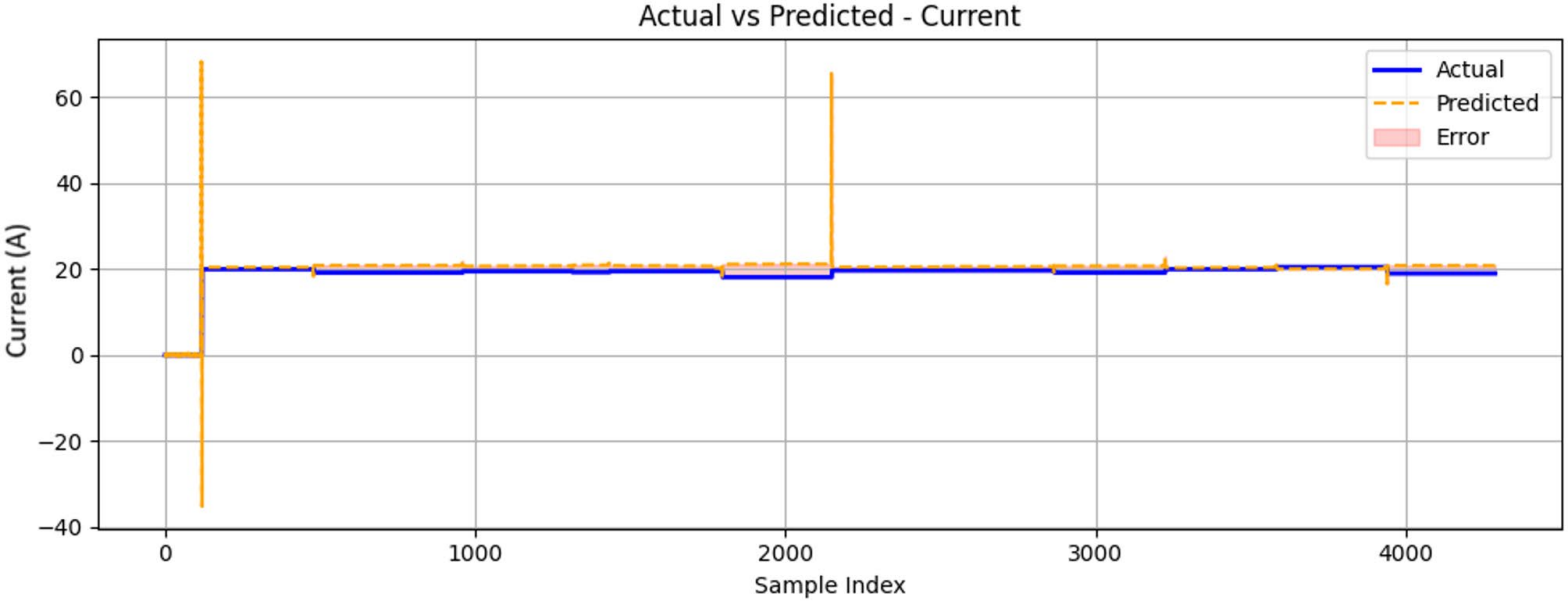
Comparison between measured and ConvLSTM2D-predicted generator output current (A) as a function of sample index during engine operation.

The current feature results shown in Fig. 23 provide a visual representation of the prediction error. The graph demonstrates that the prediction model was able to closely follow the trend of the actual current values, with some deviations indicated by the error regions. These deviations suggest areas where the model’s predictions were less accurate, which could be due to various factors such as model complexity, feature selection, or data variability. The overall performance of the model in predicting current values appeared to be satisfactory, as the predicted values generally aligned with the actual values.

**Fig. 24:**
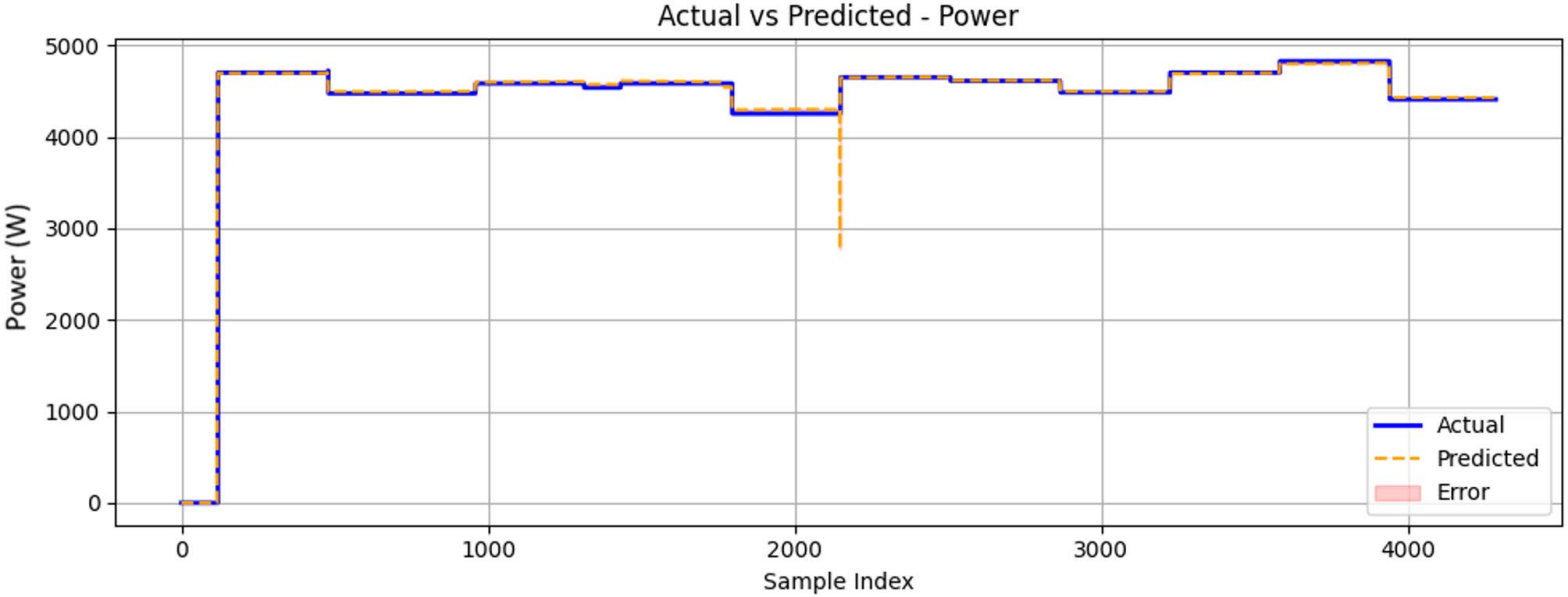
Comparison between measured and ConvLSTM2D-predicted generator output power (W) as a function of sample index during engine operation.

Figure 24 shows a comparison between the actual power values and the predicted power values for the engine. The graph demonstrates that the ML model was able to closely follow the trend of the actual power values, with some deviations indicated by the error regions. These deviations suggest areas where the model’s predictions were less accurate, which could be due to various factors such as model complexity, feature selection, or data variability. The overall performance of the ML model in predicting power values appeared to be satisfactory, as the predicted values generally align with the actual values.

**Fig. 25:**
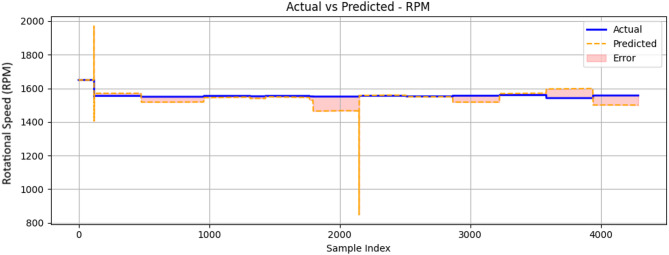
Comparison between measured and ConvLSTM2D-predicted engine rotational speed (RPM) as a function of sample index during engine operation.

Figure 25 compares the actual RPM values with the predicted RPM values generated by the ML prediction algorithm. The graph demonstrates that the ML model was able to closely follow the trend of the actual RPM values, with some deviations indicated by the error regions. The overall performance of the ML model in predicting RPM values appeared to be adequate. However, these predicted values show an underestimation of engine RPM.

**Fig. 26:**
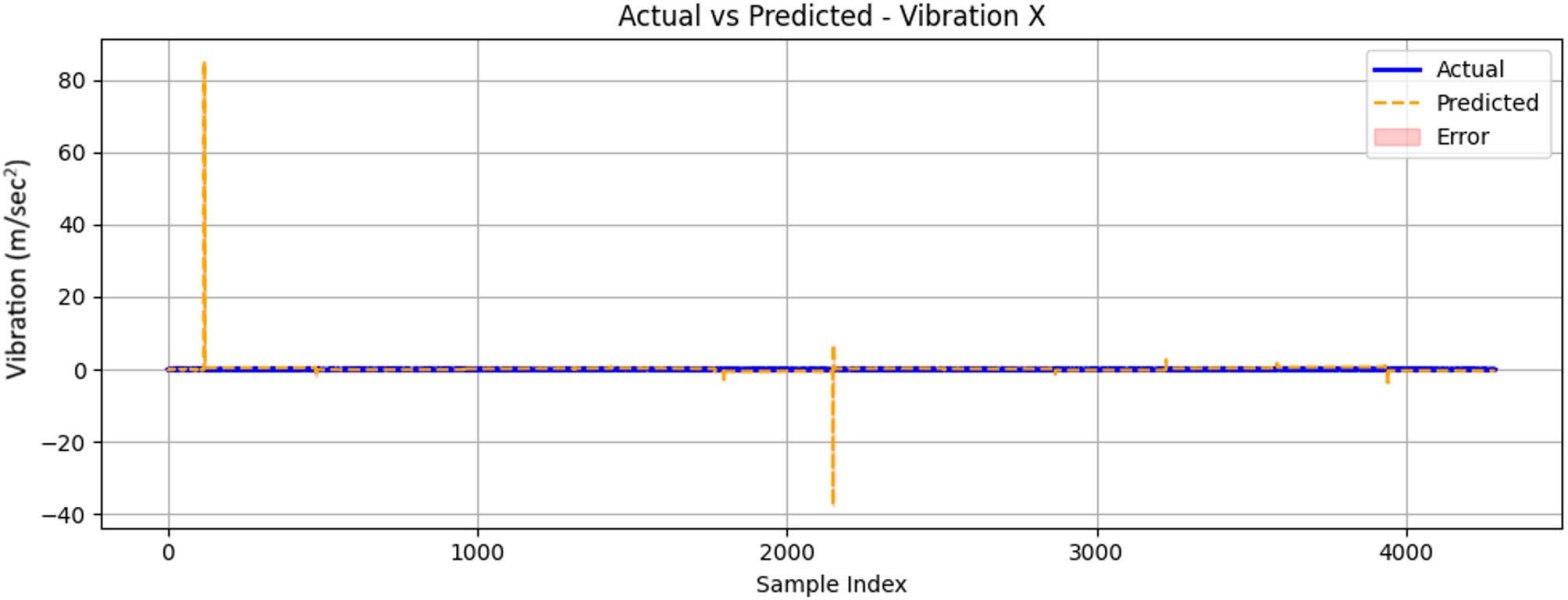
Comparison between measured and ConvLSTM2D-predicted vibration signal along the x-axis (m/sec^2^) as a function of sample index.

The results of the vibration readings in the x-direction, illustrated in Fig. 26, show areas where the model’s predictions were less accurate, particularly around sample indices approximately 1000 and 6000, where significant spikes were observed. The overall performance of the machine learning model in predicting x-axis vibration values appeared to be satisfactory. These findings highlight the potential of ML algorithms in predicting engine performance metrics, such as x-axis vibration, based on collected data. Proactive maintenance strategies can leverage these predictions to anticipate and address issues before they escalate, ensuring optimal engine performance and longevity.

**Fig. 27:**
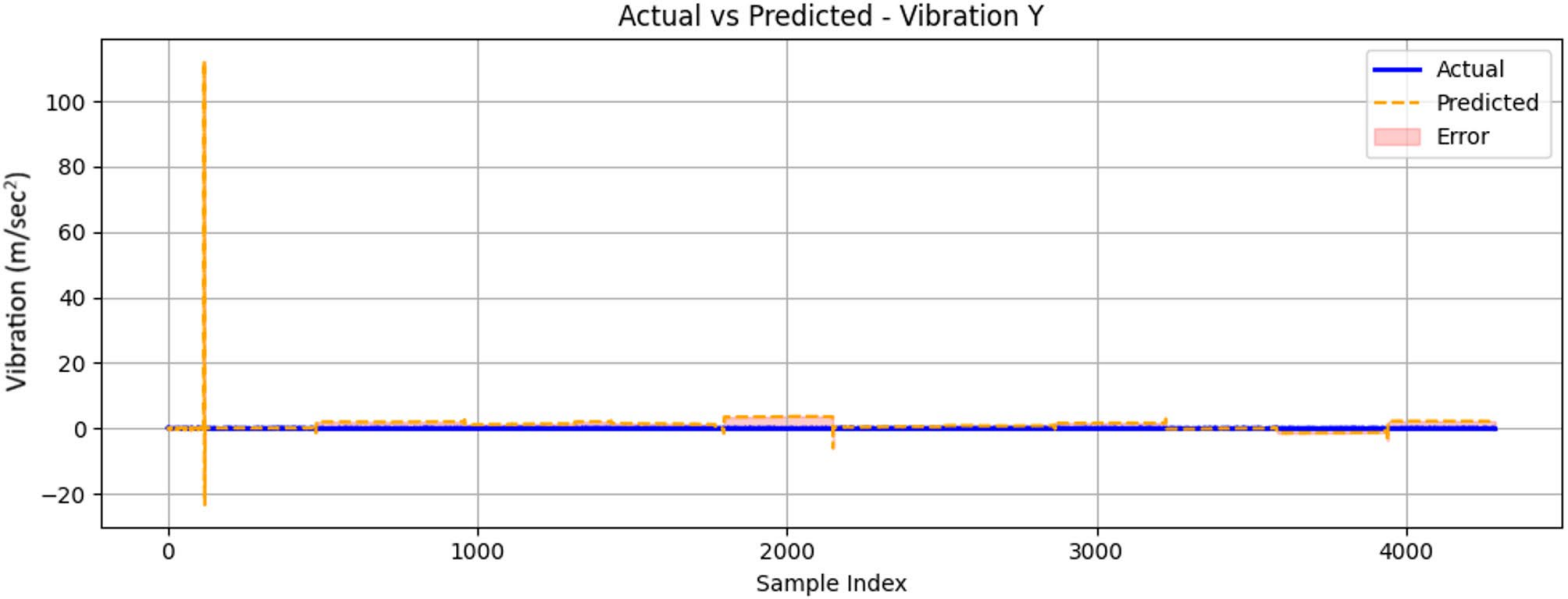
Comparison between measured and ConvLSTM2D-predicted vibration signal along the y-axis (m/sec^2^) as a function of sample index.

Figure 27 shows the vibration prediction results for the y-axis component which demonstrates the ML algorithm’s ability to capture complex mechanical behaviour under varying operational conditions. As shown in the graph, the model successfully tracked the overall trend of actual vibration measurements across the entire 4000-sample test sequence, maintaining close alignment with experimental data throughout most of the operational range. While the general waveform shape is well-preserved, closer examination showed the algorithm tended to slightly underpredict peak vibration amplitudes during transient events, particularly in the higher sample ranges (above 3000 sample index). On the other hand, the prediction accuracy appeared particularly strong in the mid-range samples (1000–3000 sample index), where the algorithm reliably followed both the amplitude and frequency characteristics of the mechanical vibrations.

**Fig. 28:**
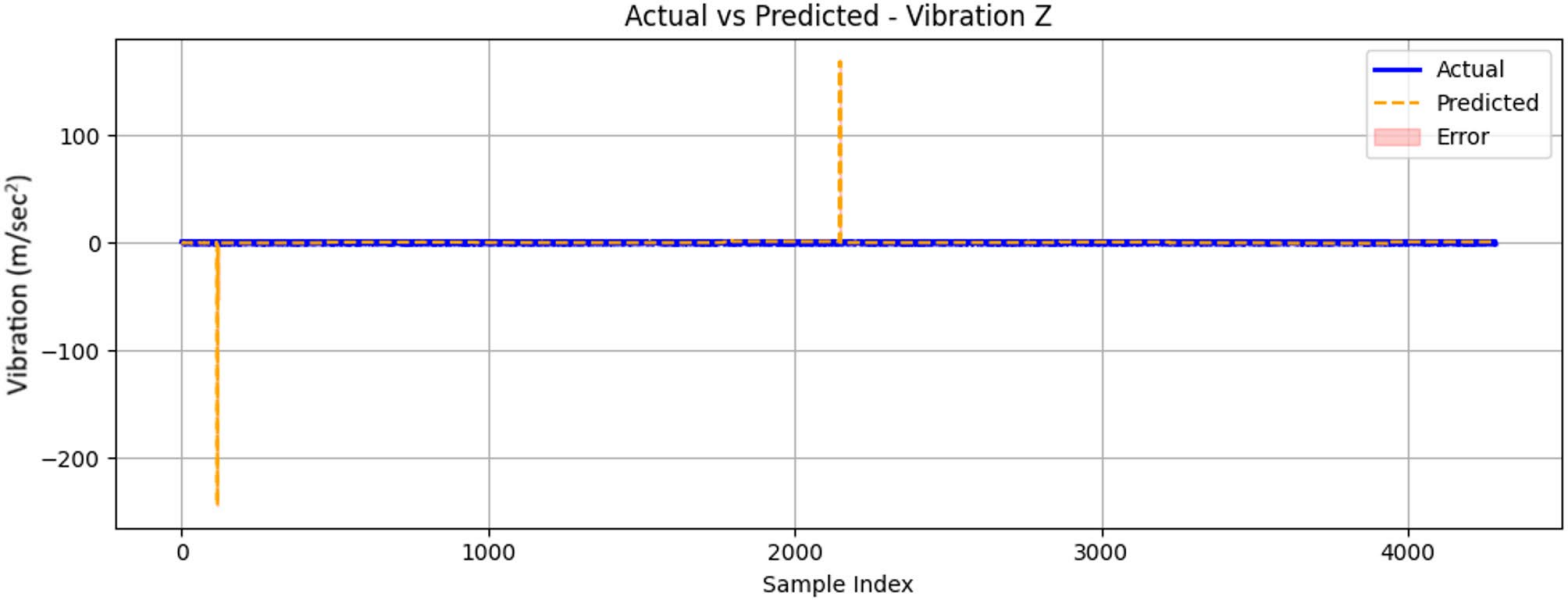
Comparison between measured and ConvLSTM2D-predicted vibration signal along the z-axis (m/sec^2^) as a function of sample index.

The comparison graph for the z-axis vibration is illustrated in Fig. 28. It shows the ML algorithm’s effectiveness in capturing the complex dynamics of vertical mechanical oscillations. Across the 4000-sample test sequence, the model showed strong agreement with experimental measurements, accurately reproducing both the amplitude and temporal characteristics of the vibration patterns. The prediction maintained particularly close alignment with actual values in the 1000–3000 sample range, where the error margin narrows significantly, indicating robust learning of the engine’s typical vibration signatures.

The prediction model yielded an MSE of 0.20044, an MAE of 0.32812, and an RMSE of 0.4477 which prove its accuracy in making future predictions for each operational mode. To further assess the performance of the model, it was compared against other prediction models such as Decision Tree Regression (DTR), ANN, and LSTM used in similar applications. All prediction models were trained and evaluated using the same datasets and test/train split, ensuring identical training, validation, and testing datasets.

The comparison results in Table 2 shows that the proposed model was capable of making time-series predictions with less deviations when compared with other models currently in use.

**Table 2:** Comparison of ML prediction models and percentage improvement of the proposed ConvLSTM2D model.

Error	Proposed	DTR	ANN	LSTM	Improvement		
					vs. DTR	vs. ANN	vs. LSTM
MSE	0.20044	0.29537	0.28035	0.52006	32.1%	28.5%	61.5%
MAE	0.32812	0.27315	0.4064	0.55499	−20.1%	19.3%	40.9%
RMSE	0.4477	0.54348	0.52948	0.721115	17.6%	15.5%	37.9%

The superior performance of the proposed ConvLSTM2D model can be attributed to the characteristics of the acquired dataset and the architectural advantages of the model. The experimental data consisted of multivariate time-series records derived from sensors. These records clearly reflected the consequential nature of the data and highlighted the transition from different levels of failure modes smoothly.

Conventional models such as DTR treat each time step independently and lack the capability to capture sequential dependencies, ANN models improve nonlinear mapping but do not model temporal correlations, which limits their effectiveness for dynamic engine behaviour. While standard LSTM networks capture long-term temporal dependencies, they operate on flattened input sequences and do not explicitly use local temporal feature correlations across the different sensor parameters.

In contrast, the ConvLSTM2D architecture integrates convolutional operations within recurrent memory units, enabling simultaneous learning of localized temporal patterns and long-range sequence dependencies. This capability is particularly important in engine monitoring data because it enhances feature extraction by maintaining its relationships among the remaining features.

Despite the strong prediction performance of the proposed ConvLSTM2D model, localized prediction errors were observed which can be attributed to several factors. First, sensor noise and measurement uncertainty, especially in vibration and temperature measurements, introduced high-frequency fluctuations that are difficult to fully capture through time-series learning alone. Second, sudden non-linear system responses, such as sudden changes in electrical load, cooling efficiency, or combustion behaviour, generated short-duration deviations from learned sequential patterns. Third, the model relied on a finite temporal window for prediction. Although sufficient for capturing most operating behaviours, limited historical data can limit the model’s ability to predict rare or delayed effects.

Several limitations of the current study should also be acknowledged despite the strong predictive performance demonstrated by the proposed ConvLSTM2D model.

First, the dataset was obtained from a controlled experimental setup using a single four-cylinder diesel engine, which limits generalization to engines of different configurations, sizes, or operating environments. Second, the fault scenarios considered in this study were restricted to a set of commonly encountered failure modes. While these failures are representative of practical engine degradation mechanisms, real-world systems may exhibit a broader range of fault types and combined failure interactions.

In addition, the dataset size, although sufficient for the present experimental validation, remains limited compared to large-scale industrial datasets typically available in fleet-level monitoring applications. The controlled laboratory conditions also reduce the influence of environmental variability. These limitations, however, do not affect from the validity of the proposed framework but define its current scope of applicability. Future work will focus on expanding the dataset, incorporating additional fault scenarios, and validating the approach under real-world operating conditions to further enhance model robustness.

### Engine failure modes classification

The classification model achieved an accuracy of 82.681%, a precision of 0.85905, a recall of 0.84907, and an f1-score of 0.84694. To further assess the performance of the model, it was compared against other classification models such as 1D CNN and MLP used in similar applications as detailed in Table 3. All classification models were trained and evaluated using the same datasets and test/train split, ensuring identical training, validation, and testing datasets.

**Table 3:** Comparison of ML classification models and percentage improvement of the proposed RF model.

Metric	Random Forest (Proposed)	1D CNN	MLP	Improvement	
				vs. 1D CNN	vs. MLP
Accuracy	0.82681	0.56037	0.32191	47.55%	156.85%
Precision	0.85905	0.61811	0.23486	38.98%	265.77%
Recall	0.84907	0.62027	0.37325	36.89%	127.48%
f1-score	0.84694	0.58396	0.26463	45.03%	220.05%

**Fig. 29:**
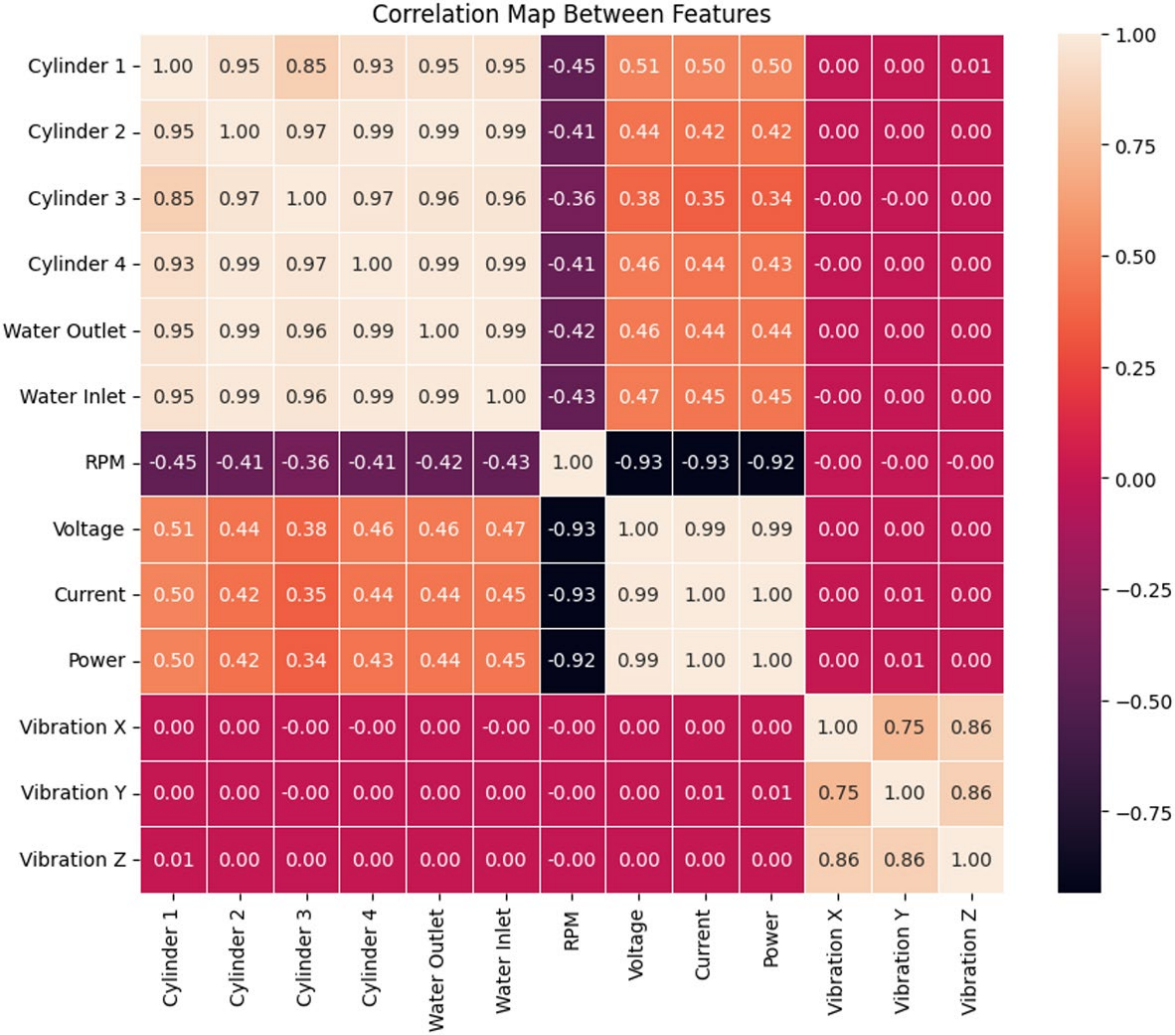
Pearson correlation matrix (dimensionless, − 1 to + 1) between the measured engine features used for model training: cylinder head temperatures for Cylinders 1–4 (°C), cooling water inlet/outlet temperatures (°C), engine speed (RPM), generator electrical features (voltage, V; current, A; power, W), and vibration signals along x/y/z axes (m/sec^2^).

Macro-averaged values of the matrix were also obtained which consider that all the proposed classes contribute to the final average quantity equally. In addition, weighted-averaged values, which show the contribution of each class to the average quantity depending on its size, were also investigated. Figure 29 illustrates the correlation matrix used to analyse relationships between features.

**Fig. 30:**
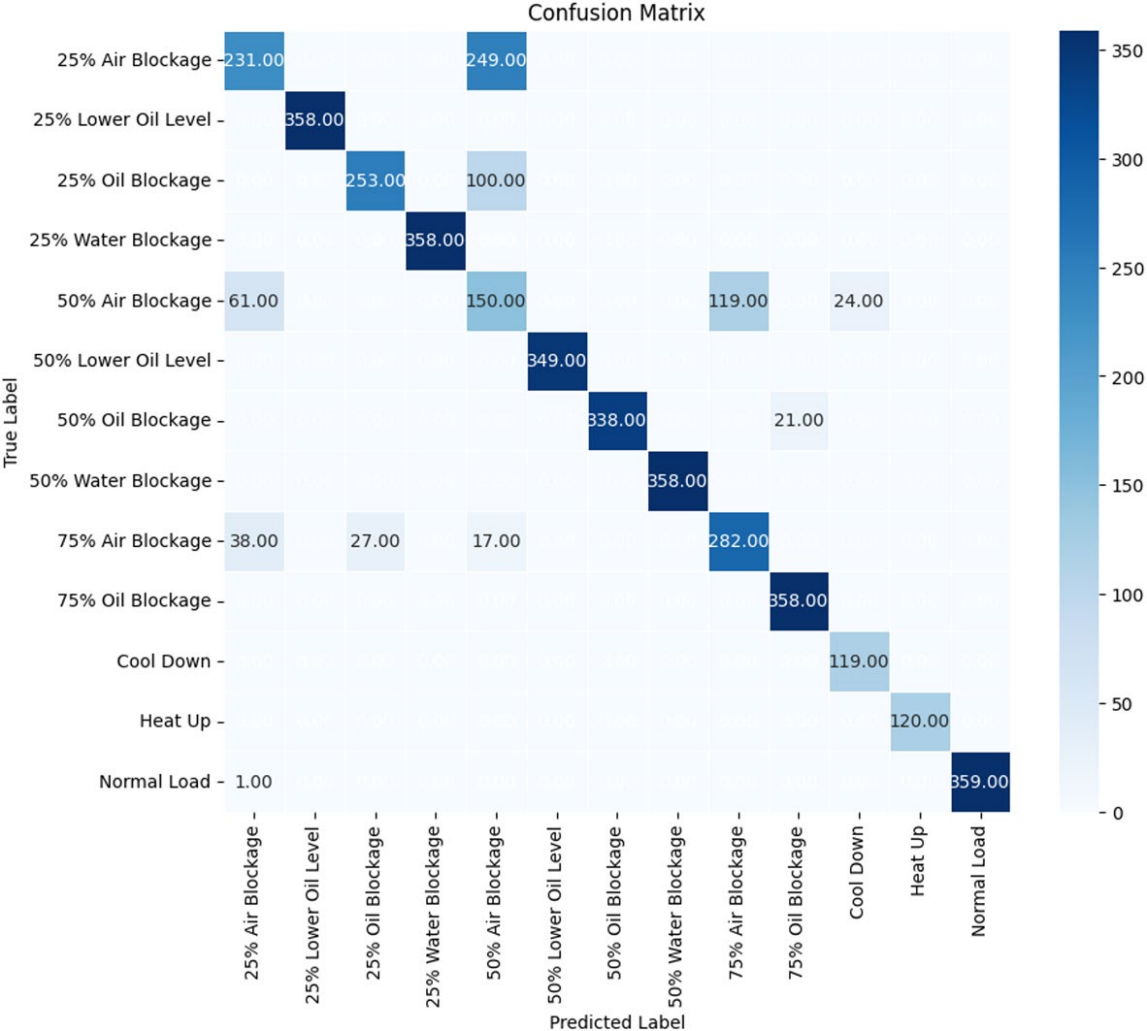
Confusion matrix for the Random Forest (RF) classification model across all operational modes and fault scenarios (counts on the test set).

Figure 30 illustrates the confusion matrix for the random forest (RF) classifier across all operational modes and imposed fault scenarios. Overall, the matrix shows strong diagonal dominance, indicating high classification accuracy for most classes; however, the off-diagonal parameters reveal that the remaining errors are not random and are concentrated in a limited set of specific, physically interpretable confusions.

The most prominent misclassification occured within the air blockage severity levels, where adjacent blockage levels partially overlap in their feature parameters. In particular, a great portion of the 25% Air Blockage samples were predicted as 50% Air Blockage, indicating that the classification class at 25% blockage approachs the lower bound of the 50% blockage level during transition segments. Similarly, a fraction of 50% Air Blockage samples was predicted as 75% Air Blockage, consistent with the observed increase in temperature readings and increasing baselines at higher blockage levels. In addition, some 75% Air Blockage samples were predicted as lower blockages, which indicates that short-duration stabilization periods reduce separability when the classifier relies on instantaneous or averaged features.

A second common confusion appears between 25% Oil Blockage and 50% Air Blockage, where a noticeable number of 25% oil blockage records were mapped to 50% air blockage. This behaviour is consistent with the experimental results, where both faults generated moderate increases in cylinder head temperature and cooling water outlet temperature, yielding overlapping thermal trends that reduce class distinctiveness. By contrast, classes associated with more distinctive temperature trends, such as 25% and 50% Water Blockage, showed diagonal concentration.

Operational modes (Heat Up, Cool Down, and Normal Load) also exhibited high separability with minimal confusion with fault classes, indicating that the model reliably distinguished transient operating conditions from failure parameters. Overall, the confusion matrix indicated that the classifier was highly effective at identifying fault type, while the majority of errors arise from differentiating close fault severities whose temperature parameters overlap.

Although the Random Forest classifier achieved an overall accuracy of approximately 82%, the adequacy of this performance for real-world use depends on the intended operational role of the system. In this study, the proposed framework is positioned as an early-warning and decision-support tool for proactive maintenance rather than as an autonomous protection system. Under this assumption, the achieved accuracy is considered suitable as a screening layer because the confusion matrix indicated that most errors occur between close failure levels within the same failure mode rather than systematic confusion between different failure types.

Nevertheless, misclassification introduces operational risk. The most critical risk is a false negative, where a failure is classified as normal operation or a failure mode with less severity, potentially delaying intervention, accelerated wear, or unplanned downtime. A second risk is a false positive, which may lead to unnecessary inspections and increased maintenance. To mitigate these risks in real-world application, several precautions are recommended such as reporting and optimizing classes precision/recall and prioritizing higher recall for safety-critical failures. These measures support safer and more reliable integration of the proposed ML framework into operational proactive maintenance workflows.

### Integrated prediction and classification

The final phase of the study involved integrating the developed prediction and classification models into one workflow. Using the trained ConvLSTM model, predictions for engine parameters were generated for 5 random scenarios obtained from the test data after the stabilization of engine operation. These predictions were subsequently fed into the RF classification model, which categorized each scenario into one of 13 predefined failure modes. To enable analysis and effective visualization, each scenario’s features were summarized (averaged) and presented in the form of radar charts.

**Fig. 31:**
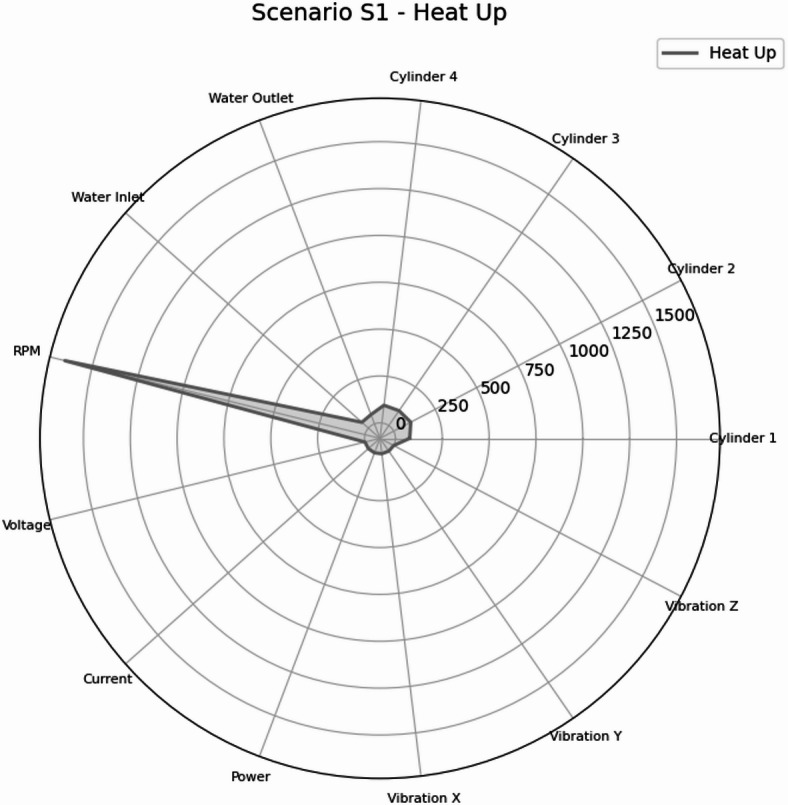
Radar plot of the mean feature values for Scenario S1 (Heat Up) across the measured parameters: cylinder head temperatures for Cylinders 1–4 (°C), cooling water inlet/outlet temperatures (°C), engine speed (RPM), generator electrical variables (voltage, V; current, A; power, W), and vibration components (x/y/z; m/sec^2^).

Figure 31 illustrates Scenario S1 which provides the baseline operating signature used to interpret deviations resulting from failure modes. The feature profile is dominated by nominal RPM and stable electrical variables, while thermal variables reflected a controlled warm-up progression without load-driven instability. This baseline is useful for maintenance logic because subsequent failure scenarios are primarily identified by alterations from this normal heat-up conditions.

**Fig. 32:**
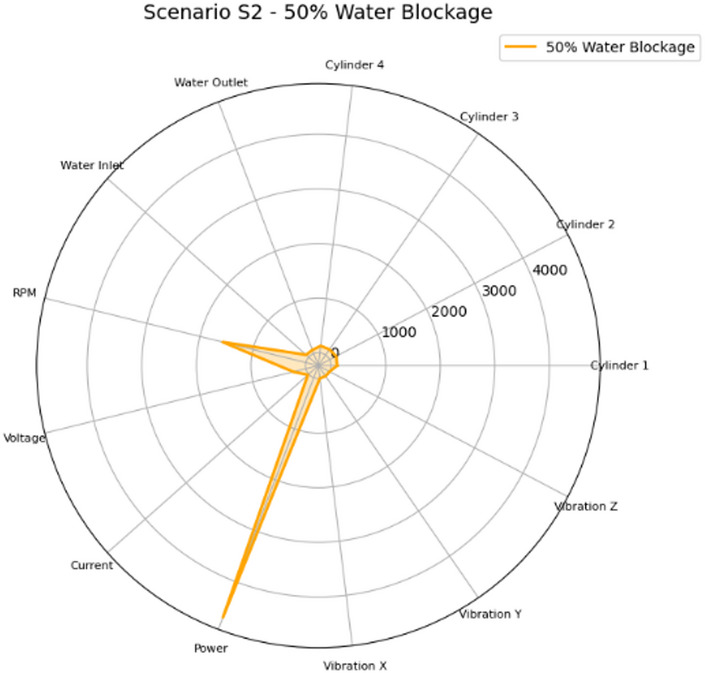
Radar plot of the mean feature values for Scenario S2 (50% Water Blockage) across the measured parameters: cylinder head temperatures for Cylinders 1–4 (°C), cooling water inlet/outlet temperatures (°C), engine speed (RPM), generator electrical variables (voltage, V; current, A; power, W), and vibration components (x/y/z; m/sec^2^).

Figure 32 shows the radar chart for Scenario S2 (50% Water Blockage) which exhibits a cooling system failure mode characterized by an elevated water outlet temperature and a clear cylinder-to-cylinder thermal gradient, reflecting reduced heat rejection and non-uniform cooling distribution. Compared with S1, disproportionate rise was found in cooling water outlet temperature relative to other operating variables. This pattern supports actionable interventions targeting the cooling water cycle before severe overheating develops.

**Fig. 33:**
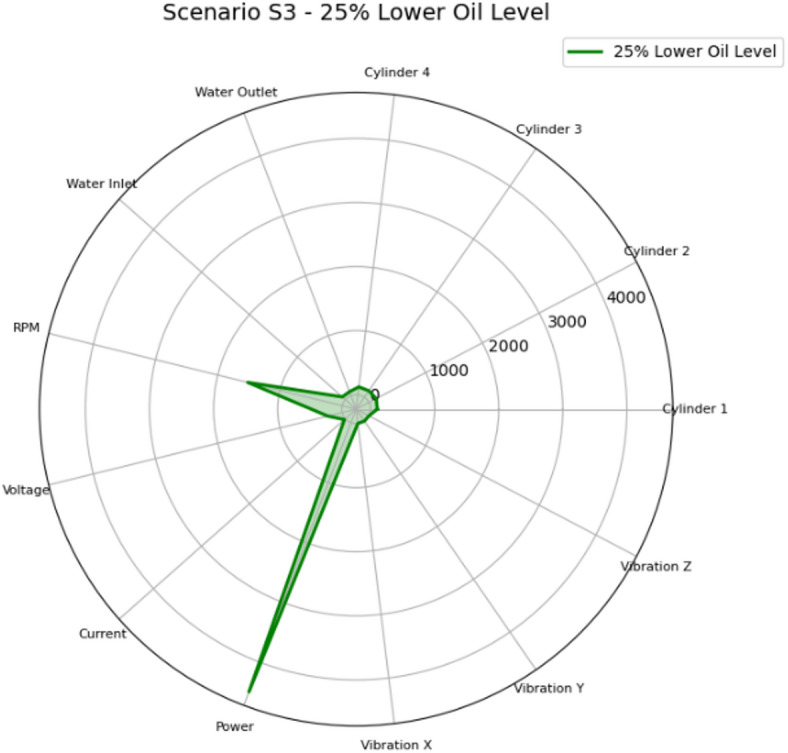
Radar plot of the mean feature values for Scenario S3 (25% Lower Oil Level) across the measured parameters: cylinder head temperatures for Cylinders 1–4 (°C), cooling water inlet/outlet temperatures (°C), engine speed (RPM), generator electrical variables (voltage, V; current, A; power, W), and vibration components (x/y/z; m/sec^2^).

The radar chart for Scenario S3 (25% Lower Oil Level) illustrated in Fig. 33 shows a multi-axis vibration increase with secondary thermal effects. Relative to S1 and S2, the failure effect shifted from coolant-temperature to friction-related vibration, consistent with reduced lubrication film thickness. Maintenance actions are therefore lubrication-centred, with vibration acting as the leading indicator and outlet temperature serving as supportive evidence of frictional heating.

**Fig. 34:**
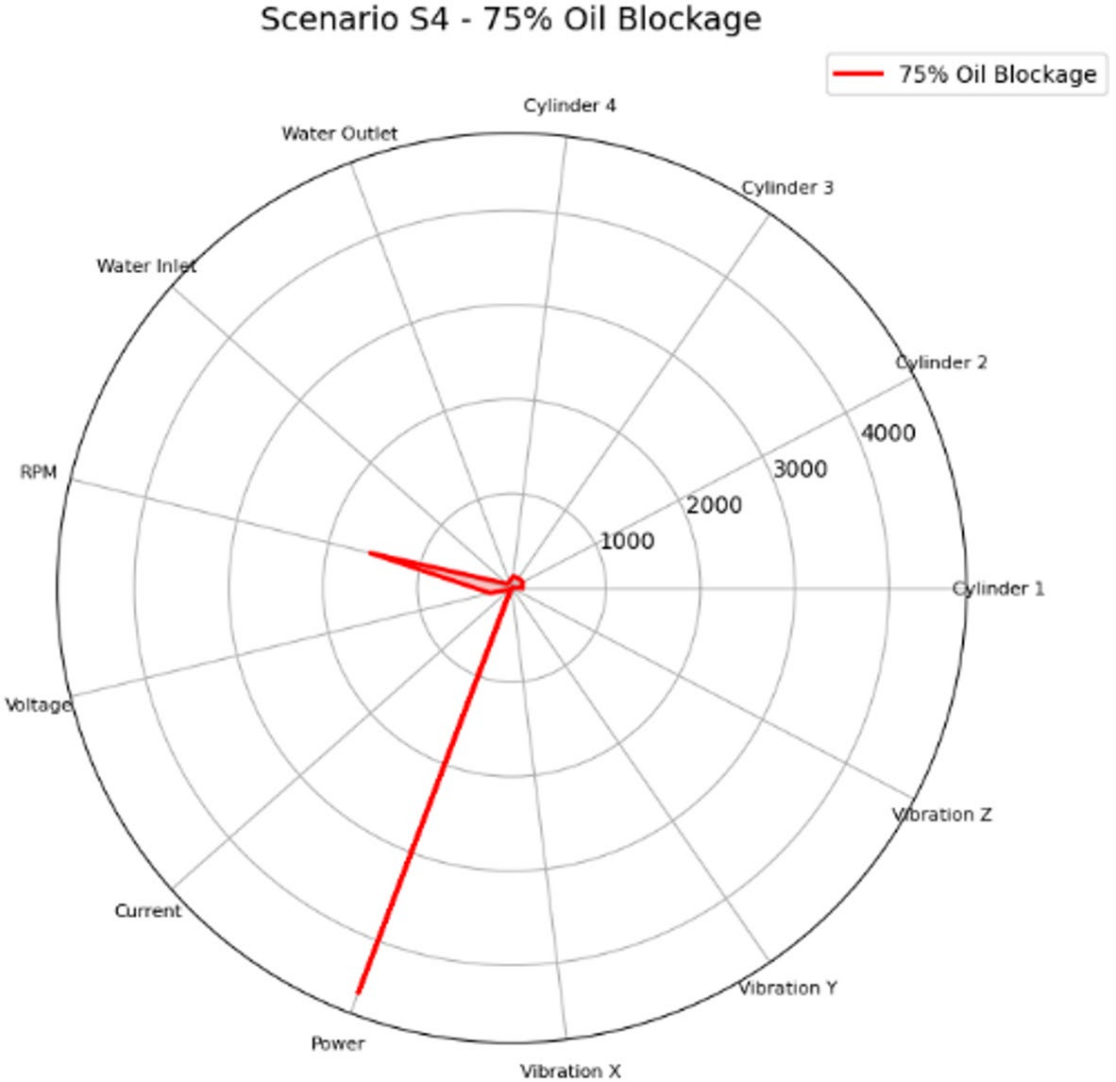
Radar plot of the mean feature values for Scenario S4 (75% Oil Blockage) across the measured parameters: cylinder head temperatures for Cylinders 1–4 (°C), cooling water inlet/outlet temperatures (°C), engine speed (RPM), generator electrical variables (voltage, V; current, A; power, W), and vibration components (x/y/z; m/sec^2^).

Figure 34 shows the radar chart for Scenario S4 (75% Oil Blockage) which represents a critical lubrication blockage failure. Compared with S3, vibration levels rose substantially and were accompanied by a strong increase in water outlet temperature, indicating severe frictional heat generation and increased cooling demand. The combination of high vibration with high water outlet temperature supports a mechanical effect and must be immediately inspected to prevent rapid wear or bearing damage.

**Fig. 35:**
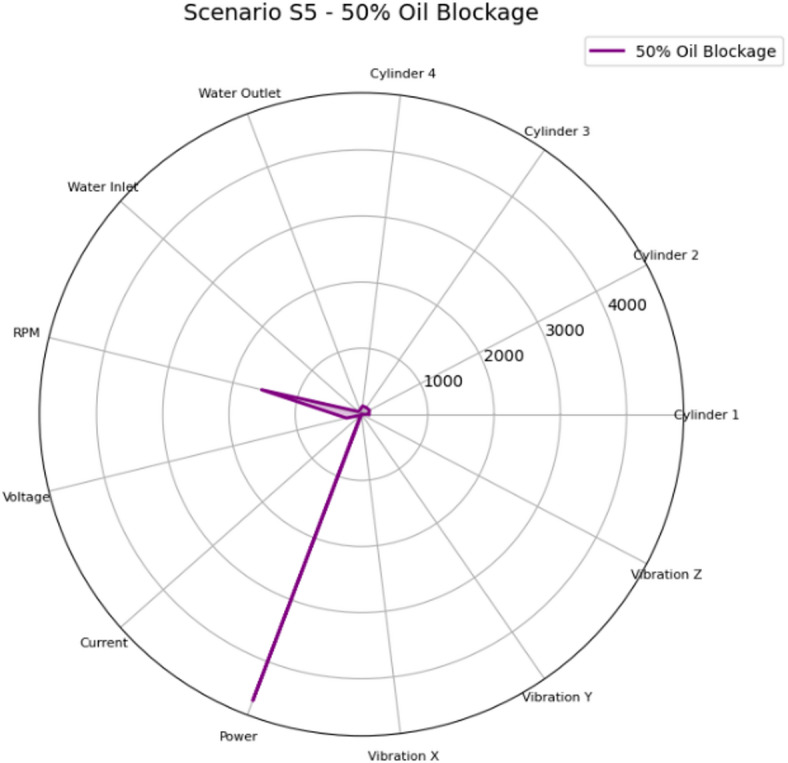
Radar plot of the mean feature values for Scenario S5 (50% Oil Blockage) across the measured parameters: cylinder head temperatures for Cylinders 1–4 (°C), cooling water inlet/outlet temperatures (°C), engine speed (RPM), generator electrical variables (voltage, V; current, A; power, W), and vibration components (x/y/z; m/sec^2^).

Scenario S5, as illustrated in Fig. 35, shows an intermediate degradation that can be placed between S3 and S4. The feature profile indicated moderate vibration elevation with a clear increase in water outlet temperature and a measurable reduction in power output, reflecting growing friction losses before reaching the critical severity seen in S4. Practically, this scenario is important for proactive maintenance because it defines a “repair window” where corrective action can be scheduled before escalation to severe blockage.

Across scenarios, the radar plots demonstrated that failure classification was driven by dominant features: cooling faults were indicated by the high temperatures in cooling water outlet (S2), whereas lubrication failures were indicated by changes in vibration along with cooling water outlet temperature rise based on failure level (S3–S5). The prediction ConvLSTM2D model provided consistent feature forecasts, and the Random Forest classifier assigned the final failure model for the predicted parameters. Residual misclassifications were expected primarily between scenarios with overlapping features, suggesting that additional sensitive inputs could further improve classification.

To support real-world proactive maintenance, the proposed framework is designed as an integrated monitoring system that combines short-term prediction with failure classification. In the proposed real-world architecture, sensors, for each measurable parameter within ships’ engine rooms, will be installed to continuously acquire data. The data will be streamed to a data acquisition unit for filtering, synchronization, and feature formatting. A rolling time window of recent measurements will then be passed to the ConvLSTM2D model to forecast future engine parameters over the next prediction window. These predicted parameters will provide an early indication of any abnormal trends before alarm thresholds are reached. The predicted features will subsequently be fed into the Random Forest classifier, which assigns the most probable operating mode/failure mode and severity level based on learned multi-parameter signatures.

This prediction–classification integrated framework will enable a proactive maintenance approach. The prediction algorithm will detect emerging deviations ahead of time while the classification algorithm will identify the most probable failure and its severity. By combining forecasting with diagnosis, the framework supports maintenance actions before faults evolve into performance degradation or shutdown events, thereby reducing unplanned downtime and avoiding catastrophic damage.

## Conclusion and future work

In this study, the behaviour of a diesel engine under normal operation and representative failure scenarios was investigated using experimental measurements and machine learning (ML) methods to support a proactive maintenance framework. The analysis considered cylinder head temperatures for Cylinders 1–4, cooling water inlet and outlet temperatures, tri-axial vibration, engine speed (RPM), and generator electrical variables (voltage, current, and power). The proposed ConvLSTM2D prediction model achieved an RMSE of 0.4477, demonstrating improved forecasting performance relative to baseline models. The Random Forest classifier achieved an overall accuracy of 82.168% in identifying operational/failure scenarios, indicating that multi-parameter feature signatures can effectively support early fault identification and maintenance decision support under controlled experimental conditions.

The results confirm that integrating forecasting (prediction of future parameters) with classification (assignment of failure modes) provides a practical proactive maintenance workflow. Prediction enables early detection of developing changes before threshold alarms are reached, while classification translates those deviations into diagnostic labels that can guide targeted inspection and corrective action. The remaining classification errors observed in the confusion matrix are primarily associated with scenarios that exhibit overlapping thermal features, highlighting the importance of adding severity-sensitive measurements and improving distinction between similar modes.

Future work will focus on improving model robustness and deployment relevance. Severity-sensitive sensors can be added to reduce ambiguity between similar failure classes. Also, oil pressure sensors (after the oil pump) and oil differential pressure across the filter to quantify lubrication blockage severity and help separate oil blockage from similar failures.

Cooling water flow rate and pressure sensors can be used to provide direct evidence of cooling-water blockages severity and improve differentiation between partial cooling water blockage levels. Furthermore, air intake manifold pressure (MAP) and intake air mass flow (MAF) sensors can be used to directly capture air blockage effects and improve accurate classification between air blockage severities. Exhaust gas temperature (EGT) per cylinder can also be utilized to strengthen combustion-related failure effects and help distinguish air-related failures from lubrication and cooling failures.

In addition, the current dataset is based on controlled experiments and defined set of failure scenarios. Future work will expand the dataset by incorporating additional fault types (e.g., injector malfunction, fuel restriction, cooling pump degradation) and broader operating conditions (variable load, ambient conditions), followed by validation on operational engines where feasible.

The proposed framework can also be implemented in a real-time framework where sensor streams are processed onboard for low-latency prediction/classification, and a cloud layer performs long-term trend analytics, fleet-level benchmarking, and maintenance scheduling. This requires secure data handling, model update strategies, and health-monitoring dashboards that communicate failure probability, severity, and recommended actions.

Overall, these targeted developments aim to enhance diagnostic specificity, reduce misclassification risk, and support practical deployment of a proactive maintenance framework that improves reliability, reduces downtime, and extends the service life of marine diesel engine components.

## Data Availability

The dataset generated in this paper is available from the corresponding author upon reasonable request.

## References

[1] U. N. United Nations Conference on Trade and Development (UNCTAD), *UNCTAD Handbook of Statistics 2023*, 1st ed. in United Nations Conference on Trade and Development (UNCTAD) Handbook of Statistics / Manuel de Statistiques de la CNUCED Series. Bloomfield: International Trade Centre (2023).

[2] United Nations Conference on Trade and Development. *Harnessing the Potential of Nutraceutical Products for Export Diversification and Development in Landlocked Developing Countries: Assessment of Comparative Advantages and Binding Constraints*. Erscheinungsort nicht ermittelbar: United Nations (2023).

[3] United Nations Conference on Trade and Development, Review of Maritime Transport 2023: Towards a Green and Just Transition. in Review of Maritime Transport. United Nations (2023). 10.18356/9789213584569.

[4] International Maritime Organization. *2023 IMO Strategy on Reduction of GHG Emissions from Ships* (2023). https://www.imo.org/en/ourwork/environment/pages/2023-imo-strategy-on-reduction-of-ghg-emissions-from-ships.aspx.

[5] Duran, V., Uriondo, Z. & Moreno-Gutiérrez, J. The impact of marine engine operation and maintenance on emissions. *Transp. Res. Part. Transp. Environ.* **17** (1), 54–60. 10.1016/j.trd.2011.09.001 (2012).

[6] Vaisnys, P., & Contri, P. *Monitoring the Effectiveness of Maintenance Programmes Through Use of Performance Indicators*. Bruxelles (Belgium): European Nuclear Society (2007). http://www.euronuclear.org/events/enc/enc2007/pdf/ENC2007_programme.pdf.

[7] Dadash, A. H. & Björsell, N. Effective machine lifespan management using determined state–action cost estimation for multi-dimensional cost function optimization. *Prod. Manuf. Res.* **12** (1), 2383656. 10.1080/21693277.2024.2383656 (2024).

[8] University of Strathclyde, Glasgow, U. K., Turan, O. & Lazakis, I. University of Strathclyde, Glasgow, UK, S. Judah, and Scottish and Southern Energy Renewables (SSER), Glasgow, UK, Establishing the Optimum Vessel Maintenance Approach Based on System Reliability and Criticality Analysis. In *Managing Reliability & Maintainability in the Maritime Industry, RINA* (2012). 10.3940/rina.rm.2012.04.

[9] Leukel, J., González, J. & Riekert, M. Adoption of machine learning technology for failure prediction in industrial maintenance: a systematic review. *J. Manuf. Syst.* **61**, 87–96. 10.1016/j.jmsy.2021.08.012 (2021).

[10] Saihi, A., Ben-Daya, M. & As’ad, R. A. Maintenance and sustainability: a systematic review of modeling-based literature. *J. Qual. Maint. Eng.* **29**, 155–187. 10.1108/JQME-07-2021-0058 (2023).

[11] Ventikos, N. P., Sotiralis, P. & Annetis, E. A combined risk-based and condition monitoring approach: developing a dynamic model for the case of marine engine lubrication. *Transp. Saf. Environ.* **4** (3), tdac020. 10.1093/tse/tdac020 (2022).

[12] Lapa, C. M. F., Pereira, C. M. N. A. & De Barros, M. P. A model for preventive maintenance planning by genetic algorithms based in cost and reliability. *Reliab. Eng. Syst. Saf.* **91**, 233–240. 10.1016/j.ress.2005.01.004 (2006).

[13] Simion, D., Purcarea, A., Cotorcea, A., Florin, N. & Cosofret, D. Naval maintenance. From corrective maintenance to condition monitoring and IoT. Future trends set by latest IMO amendments and autonomous ships (2021).

[14] Lazakis, I., Turan, O. & Aksu, S. Improving ship maintenance: a criticality and reliability approach. In *11th International Symposium on Practical Design of Ships and Other Floating Structures, PRADS 2010* (2010).

[15] Karatuğ, Ç., Arslanoğlu, Y. & Soares, C. G. Review of maintenance strategies for ship machinery systems. *J. Mar. Eng. Technol.* **22**, 233–247. 10.1080/20464177.2023.2180831 (2023).

[16] Shan, C., Chin, C. S., Mohan, V. & Zhang, C. Review of various machine learning approaches for predicting parameters of lithium-ion batteries in electric vehicles. *Batteries* **10** (6), 181. 10.3390/batteries10060181 (2024).

[17] Koilo, V. Sustainability issues in maritime transport and main challenges of the shipping industry. *Environ. Econ.* **10** (1), 48–65. 10.21511/ee.10(1).2019.04 (2019).

[18] Sujesh, G. & Ramesh, S. Modeling and control of diesel engines: a systematic review. *Alex Eng. J.* **57** (4), 4033–4048. 10.1016/j.aej.2018.02.011 ( 2018).

[19] Maione, F., Lino, P., Maione, G. & Giannino, G. A machine learning framework for condition-based maintenance of marine diesel engines: a case study. *Algorithms* **17**, 411. 10.3390/a17090411 (2024).

[20] Ingemarsdotter, E., Kambanou, M. L., Jamsin, E., Sakao, T. & Balkenende, R. Challenges and solutions in condition-based maintenance implementation - a multiple case study. *J. Clean. Prod.* **296**, 126420. 10.1016/j.jclepro.2021.126420 ( 2021).

[21] Wang, R., Chen, H. & Guan, C. A Bayesian inference-based approach for performance prognostics towards uncertainty quantification and its applications on the marine diesel engine. *ISA Trans.* **118**, 159–173. 10.1016/j.isatra.2021.02.024 (2021). 10.1016/j.isatra.2021.02.024 33642029

[22] Kalafatelis, A. S., Levis, G., Giannopoulos, A., Tsoulakos, N. & Trakadas, P. Explainable predictive maintenance of marine engines using a hybrid bilstm-attention-kolmogorov arnold network. *J. Mar. Sci. Eng.* **14** (1), 32. 10.3390/jmse14010032 (2025).

[23] Kalafatelis, A. S. et al. Towards predictive maintenance in the maritime industry: a component-based overview. *J. Mar. Sci. Eng.* **13** (3), 425. 10.3390/jmse13030425 (2025).

[24] Kirketerp-Møller, T., Hyldgaard, M. W., Cai, J., Dodis, A. I. & Rytter, N. G. M. Data-driven predictive maintenance for two-stroke marine diesel engines using machine learning and MLOps. *J. Ocean Eng. Sci.* **11**, 278–296. 10.1016/j.joes.2025.11.011 (2026).

[25] Payette, M. & Abdul-Nour, G. Machine learning applications for reliability engineering: a review. *Sustainability* **15** (7), 6270. 10.3390/su15076270 (2023).

[26] Bokonda, P. L., Ouazzani-Touhami, K. & Souissi, N. Predictive analysis using machine learning: review of trends and methods. In *2020 International Symposium on Advanced Electrical and Communication Technologies (ISAECT) Marrakech, Morocco* 1–6 (IEEE, 2020). 10.1109/ISAECT50560.2020.9523703.

[27] Krishnamurthi, R. et al. An overview of IoT sensor data processing, fusion, and analysis. *Sensors* **20**, 6076. 10.3390/s20216076 (2020). 10.3390/s20216076 PMC7663157 33114594

[28] Alshamrani, M. IoT and artificial intelligence implementations for remote healthcare monitoring systems: a survey. *J. King Saud Univ. - Comput. Inf. Sci.* **34** (8), 4687–4701. 10.1016/j.jksuci.2021.06.005 (2022).

[29] Afefy, I. H. Reliability-centered maintenance methodology and application: a case study. *Engineering* **02** (11), 863–873. 10.4236/eng.2010.211109 (2010).

[30] Eriksen, S., Utne, I. B. & Lützen, M. An RCM approach for assessing reliability challenges and maintenance needs of unmanned cargo ships. *Reliab. Eng. Syst. Saf.* **210**, 107550. 10.1016/j.ress.2021.107550 (2021).

[31] Jimenez, V. J., Bouhmala, N. & Gausdal, A. H. Developing a predictive maintenance model for vessel machinery. *J. Ocean. Eng. Sci.* **5** (4), 358–386. 10.1016/j.joes.2020.03.003 (2020).

[32] Lion, S. et al. Thermodynamic analysis of waste heat recovery using Organic Rankine Cycle (ORC) for a two-stroke low speed marine Diesel engine in IMO Tier II and Tier III operation. *Energy* **183**, 48–60. 10.1016/j.energy.2019.06.123 (2019).

[33] Rodriguez, C. G., Lamas, M. I., Rodriguez, J. D. & Abbas, A. Analysis of the pre-injection system of a marine diesel engine through multiple-criteria decision-making and artificial neural networks. *Pol. Marit. Res.* **28**, 88–96. 10.2478/pomr-2021-0051 (2022).

[34] Fernoaga, V., Sandu, V. & Balan, T. Artificial intelligence for the prediction of exhaust back pressure effect on the performance of diesel engines. *Appl. Sci.* **10**, 7370. 10.3390/app10207370 (2020).

[35] Munim, Z. H., Dushenko, M., Jimenez, V. J., Shakil, M. H. & Imset, M. Big data and artificial intelligence in the maritime industry: a bibliometric review and future research directions. *Marit. Policy Manag.* **47**, 577–597. 10.1080/03088839.2020.1788731 (2020).

[36] Tian, Z., Liu, F., Li, Z., Malekian, R. & Xie, Y. The development of key technologies in applications of vessels connected to the internet. *Symmetry* **9** (10), 211. 10.3390/sym9100211 (2017).

[37] Krakowski, R. Diagnosis modern systems of marine diesel engine. *J. KONES Powertrain Transp.*, **21**, 191–198. 10.5604/12314005.1133203 (2014).

[38] Kiritsi, A., Fountis, A. & Alkhafaji, M. A. Overview of applications of artificial intelligence methods in propulsion efficiency optimization of LNG fueled ships. In *Proceedings of 3rd International Conference on Mathematical Modeling and Computational Science, dvances in Intelligent Systems and Computing* (eds. Peng, S.-L. et al.) 391–406 (Springer Nature Singapore, 2023). 10.1007/978-981-99-3611-3_32.

[39] Menda, V. R. et al. Machine learning based prediction of the performance and emission characteristics of CRDI diesel engine using diethyl ether and carbon nanotube additives with Spirulina platensis as a third-generation biofuel. *Sci. Rep.* **15**, 39958. 10.1038/s41598-025-23747-9 (2025). 10.1038/s41598-025-23747-9 PMC12618528 41238642

[40] Koten, H. & Namar, M. M. Artificial intelligence in diesel engines. In *Diesel Engines - Current Challenges and Future Perspectives* (IntechOpen, 2024). 10.5772/intechopen.1003741.

[41] Bikkavolu, J. R. et al. Unveiling the role of nanoparticles in biodiesel blends: a comprehensive energy‐exergy‐sustainability analysis for CI engine optimization. *Energy Sci. Eng.* **13**, 6383–6399. 10.1002/ese3.70324 (2025).

[42] Viana, D. P. et al. Diesel engine fault prediction using artificial intelligence regression methods. *Machines* **11** (5), 530. 10.3390/machines11050530 (2023).

[43] Mylapalli, S. et al. Machine learning based analysis of diesel engine performance using Fe2O4 nanoadditive in sterculia foetida biodiesel blend. *Sci. Rep.* **15** (1), 39028. 10.1038/s41598-025-26178-8 (2025). 10.1038/s41598-025-26178-8 PMC12594825 41203794

[44] Bikkavolu, J. R. et al. Predicting Common Rail Direct Injection (CRDI) engine metrics using nanoparticle-enhanced pongamia pinnata biodiesel with machine learning. *Emergent Mater. Jul*. 10.1007/s42247-025-01175-9 (2025).

[45] Hickenbottom, C. Proactive approaches for engine health management and a high value example. In *2022 IEEE Aerospace Conference (AERO), Big Sky, MT, USA: IEEE* 1–6 (2022). 10.1109/AERO53065.2022.9843255.

[46] Michel, M. et al. Development of proactive maintenance plan for identification of ship’s main engine failures. In *Presented at the International Maritime Transport and Logistics Conference* 603–612 (2024). https://www.scopus.com/inward/record.uri?eid=2-s2.0-105015560234&partnerID=40&md5=f05d3e2872b68f7a6fbefe632314b591.

[47] Singh, R. et al. Facial expression recognition in videos using hybrid CNN & ConvLSTM. *Int. J. Inf. Technol.* **15**, 1819–1830. 10.1007/s41870-023-01183-0 (2023). 10.1007/s41870-023-01183-0 PMC10028317 37256027

[48] Tan, P. N., Steinbach, M., Karpatne, A. & Kumar, V. *Introduction to Data Mining* (Pearson, 2020).

